# Tobacco as an efficient metal accumulator

**DOI:** 10.1007/s10534-022-00431-3

**Published:** 2022-09-12

**Authors:** Katarzyna Kozak, Danuta Maria Antosiewicz

**Affiliations:** Department of Plant Metal Homeostasis, Faculty of Biology, Institute of Experimental Plant Biology and Biotechnology, University of Warsaw, 1 Miecznikowa Str, 02-096 Warszawa, Poland

**Keywords:** Tobacco, Phytoremediation, Zinc, Cadmium, Metal transporters

## Abstract

Tobacco (*Nicotiana tabacum* L.) is an important industrial crop plant. However, it efficiently accumulates metals, primarily cadmium (Cd) and also zinc (Zn), in its leaves. Therefore, it could be a source of cadmium intake by smokers. On the other hand, as a high leaf metal accumulator, it is widely used for phytoremediation of metal-contaminated soil. Both issues provide an important rationale for investigating the processes regulating metal homeostasis in tobacco. This work summarizes the results of research to date on the understanding of the molecular mechanisms determining the effective uptake of Zn and Cd, their translocation into shoots and accumulation in leaves. It also discusses the current state of research to improve the phytoremediation properties of tobacco through genetic modification and to limit leaf Cd content for the tobacco industry.

**Graphical Abstract:**

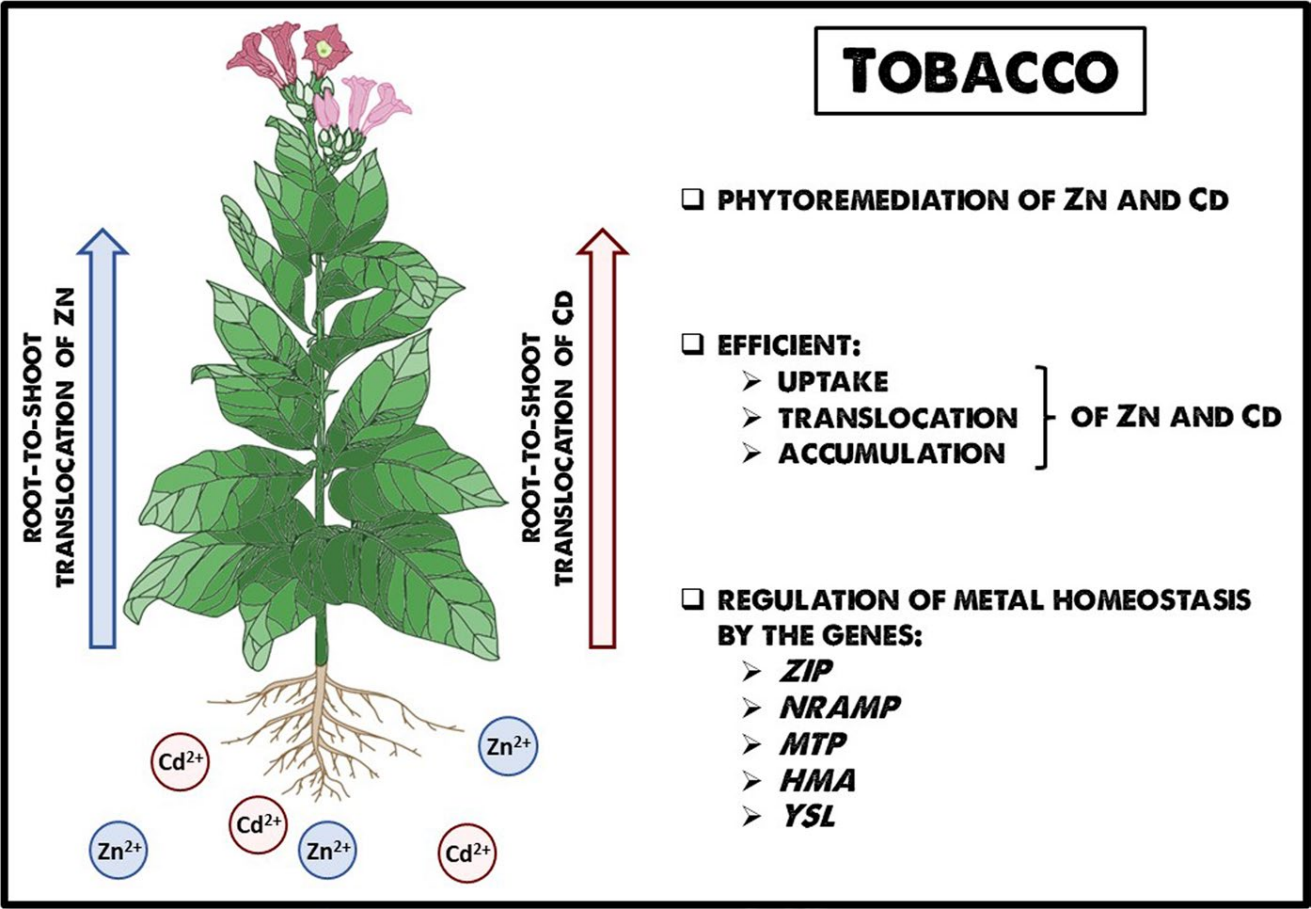

**Supplementary Information:**

The online version contains supplementary material available at 10.1007/s10534-022-00431-3.

## Introduction

Tobacco (*Nicotiana tabacum* L.) is a crop plant of great economic importance used in the production of cigarettes. However, unlike other crops and wild plants, most tobacco species are efficient leaf cadmium (Cd), and zinc (Zn) accumulators, although they differ greatly in accumulation ability (Wagner and Yeargan 1986; Angelova et al. 2004; Doroszewska and Berbeć 2004; Lugon-Moulin et al. 2004; Willers et al. 2005; Jarup and Akesson 2009; Vasiliadou and Dordas 2009; Tang et al. 2017; Kinay et al. 2018; 2021). The efficiency of accumulating Cd and Zn in tobacco leaves is so high, that their metal level frequently exceeds that of the soil/medium, which is typical of hyperaccumulators (McGrath and Zhao 2003). For example, tobacco hyperaccumulation responses were demonstrated in hydroponic experiments showing tobacco’s ability to store Cd and Zn in leaves in concentrations higher than those in the medium (Vera-Estrella et al. 2017). Similarly, Tang et al. (2017) reported a very high bioconcentration factor, BF (shoot Cd concentration to soil Cd concentration ratio), for tobacco grown on moderately contaminated agricultural soil containing 0.56 mg Cd kg^−1^ (the average Cd concentration in agricultural soil is 0.53 mg kg^−1^) from Qiyang Hunan province, China. In the Jinying and Komotini Basma cultivars, the Cd BFs were 10.1 and 17.3, respectively.

The value of the metal bioconcentration factor depends on the tobacco variety and metal concentration in the soil/nutrient solution. However, it is higher than in most non-hyperaccumulating plants, indicating effective root-to-shoot translocation. This feature makes tobacco a potential source of Cd intake for smokers. Even small amounts of Cd transferred to shoots and accumulated in leaves lead to a gradual accumulation of this harmful metal in the body of smokers. It was shown that they accumulate approximately 2-fold more Cd than non-smokers (Phu-Lich et al. 1990; Tsadilas et al. 2005; Verma et al. 2010; He et al. 2020).

There is, however, the “other side of the coin”. The ability of tobacco for high accumulation of Zn and Cd in leaves, although unfavourable to smokers, determines its usefulness for other purposes, such as phytoremediation of metal-contaminated soil (Kumar et al. 1995; Liu et al. 2011; Herzig et al. 2014).

Taking into account the wide use of tobacco plants, it is important to understand the molecular mechanisms that regulate the ability to efficiently take up and accumulate high amounts of Zn and Cd preferentially in leaves. This is of primary importance due to the signifcance of applying these results to engineering plants being more effective in phytoremediation or having lower cadmium levels in leaves used to produce cigarettes, for example. Therefore, here we summarize the current knowledge about the regulation of key processes: Zn and Cd uptake, translocation to shoots, and accumulation (which includes a range of metal homeostasis processes). We also present the scope of genetic modifications carried out, on the one hand, to increase the usefulness of tobacco for phytoextraction of Zn/Cd from contaminated soil, and on the other, to lower the Cd content of leaves (which is important for the tobacco industry).

**Fig. 1:**
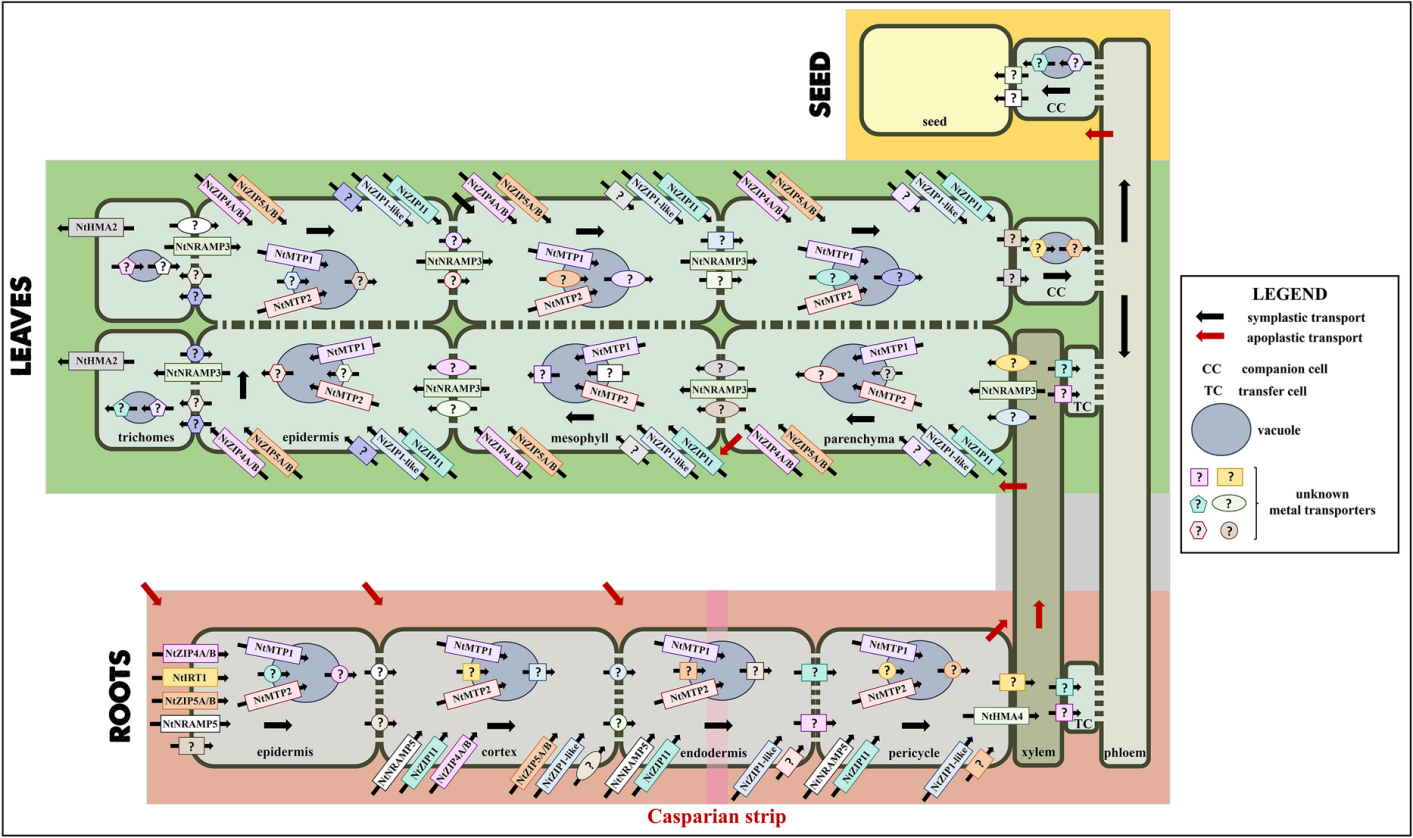
A schematic presentation of metal transporters involved in uptake, translocation and sequestration of metals in tobacco. In roots—Metals are taken up from the soil solution by NtIRT1, NtZIP4A/B, NtZIP5A/B and NtNRAMP5. In addition, NtZIP4A/B, NtZIP5A/B, NtZIP11, NtZIP1-like and NtNRAMP5 are also responsible for metal uptake by cells of internal tissues. NtMTP1 and NtMTP2 contribute to metal sequestration in the vacuoles. NtHMA4 delivers metals to the xylem. In leaves—NtZIP1-like, NtZIP4A/B, NtZIP5A/B, NtZIP11, NtNRAMP3 are involved in the uptake of metals by leaf cells. NtMTP1 and NtMTP2 contribute to metal sequestration in the vacuoles. NtHMA2 participates in metal excretion from trichomes. Metal transporters are indicated as circles, rectangulars, squares, pentagons, hexagons and ellipses with thin black arrows, unknown metal transporters are indicated with “?”. Red arrows indicate apoplastic transport. Black arrows indicate symplastic transport. CC companion cells, TC transfer cells. A large blue circle indicates a vacuole. (Color figure online)

A schematic presentation of the molecular metal transport systems in tobacco that include transporters involved in uptake, translocation and sequestration of metals is shown in Fig. 1 and Supplementary Table S1. Tobacco is an allotetraploid species having two ancestors (*N*. *sylvestris* and *N. tomentosiformis*), therefore, two gene copies originating from each of them were sometimes detected.

## Regulation of Zn and Cd homeostasis in tobacco

### Zn and Cd uptake

Zn and Cd are mainly taken up as divalent cations, although in grasses, uptake of Zn-PS (phytosiderophores) complexes also occurs (Hacisalihoglu and Kochian. 2003; Amini et al. 2021). The role of a range of other factors in the efficiency of metal uptake, such as rhizosphere acidification, root exudation, and soil characteristics (pH, organic matter content, clay content and others) in the efficiency of metal uptake is beyond the scope of this review.

Uptake of minerals by roots is accomplished through selective transporters localized at the plasma membrane. Members of the ZIP (ZRT1/IRT1-related Protein) family represent proteins that transport of a range of metal ions, including Zn and Cd, towards the cytoplasm (Vatansever et al. 2016; Li et al. 2021). Those targeted to the plasma membrane are uptake proteins. So far, two proteins involved in the uptake of zinc and cadmium by the roots directly from the soil solution have been identified. These include NtZIP4 and NtZIP5 from ZIP family (Barabasz et al. 2019; Palusińska et al. 2020). The genes encoding both transporters are present in two copies, *NtZIP4A/B* and *NtZIP5A/B*, with high homology at the protein level (97% and 98% respectively) and a with similar expression pattern. Expression of *promNtZIP4B::GUS* and *promNtZIP5B::GUS* takes place in the epidermis of the middle part of the roots. However, NtZIP4 and NtZIP5 are also involved in delivering metals to the cells of internal tissues, both in the roots and in the leaves, with different tissue specificity. Both genes are upregulated by Zn deficiency, therefore, they were considered key players in a plant’s adaptation to low Zn. Their promoters contain ZDRE elements (Zinc Deficiency Response Elements; Assunçäo et al. 2010) important for upregulation under Zn-limiting conditions. However, the role of *NtZIP5A/B* in regulating tobacco’s response to Zn deficit seems to be more specific than *NtZIP4A/B*, since under control conditions its expression is almost undetectable.

The difference in the physiological function between NtZIP4A/B and NtZIP5A/B is also due to the fact that they supply distinct tissues with Zn (Barabasz et al 2019; Palusińska et al. 2020). In the root apex, NtZIP5 mediates the uptake of metals in all meristematic tissues (except the quiescent centre), while NtZIP4 only in the procambium. In mature, middle root parts, both NtZIP4 and NtZIP5 participate in the uptake of metals from the soil solution (expression in the epidermis), but NtZIP4 plays a more universal role, as its expression can be found not only in the epidermis but also in other tissues such as the cortex and the central cylinder. Interestingly, high activity of the *NtZIP4B* promoter in the epidermis of the middle root parts detected under Zn-deficiency, disappears in the presence of Cd and upon an increased Zn level (Maślińska-Gromadka et al. 2021). It is believed that its downregulation under high metal supply may contribute to a reduction in the influx of cadmium and also to the excess of Zn. Consequently, it seems that NtZIP4A/B play a dual role in the regulation of metal homeostasis in tobacco. On one hand, at Zn-limiting conditions it participates in ensuring its good supply in the cells. On the other, at excess Cd and Zn it contributes to the limitation of metal uptake, which in tobacco is indicative of a protective mechanism against metal toxicity. Furthermore, this effect is enhanced after exposure to cadmium or zinc excess by shifting the expression site of *promNtZIP4B::GUS* from the meristematic cells of the root apex to the young differentiated tissues present within the adjacent area just above. In the leaves, both proteins are involved in the uptake of metals preferentially by palisade parenchyma (Barabasz et al 2019; Palusińska et al. 2020; Maślińska-Gromadka et al. 2021).

In many plant species, IRT1 is one of the best-known transporters responsible in the roots for absorption of both Zn and Cd from the soil solution, although its main substrate is iron (Fe) (Korshunova et al. 1999; Palmer and Guerinot 2009; Ricachenevsky et al. 2015). Although *NtIRT1* has been already cloned in tobacco (Hodoshima et al. 2007), so far only studies with the use of yeast double mutant *fet3fet4* have been performed, and indicated Fe as a substrate for the protein. However, *NtIRT1* responds to the Fe-, Zn- and Cd-status with an increase in expression at Fe-deficiency, in the presence of Cd (Yoshihara et al. 2006; Enomoto et al. 2007; Hodoshima et al. 2007), and at Zn deficiency, specifically in the apical root parts (Palusińska et al. 2020). Interesting comparative studies on the expression of *NtIRT1* between two tobacco species were performed by Bovet et al. (2006). He showed that different Cd patterns of root/shoot distribution were accompanied by distinct *NtIRT1* expression. In *N. tabacum* (Cd leaf accumulator) its constitutive transcript level (at control conditions) in the roots was low, but was induced in the presence of 1 µM Cd. In contrast, the *NtIRT1* constitutive level in *N. rustica* (Cd root accumulator) was high and decreased at exposure to Cd, suggesting different pathways of Cd uptake, transport and sequestration between species exhibiting its differential expression. Taken together, these results point to an important role for NtIRT1 in regulating tobacco responses to a range of Zn and Cd supply, but detailed studies based on yeast growth tests are needed to determine if they are substrates for this protein.

In tobacco, two other Zn (but not Fe or Cd) plasma membrane uptake transporters were identified, NtZIP1-like and NtZIP11 (Papierniak et al. 2018; Kozak et al. 2019). However, they were implicated in the uptake of Zn by the cells of internal tissues (no expression in the epidermis). Both genes differ in their expression pattern. First, *NtZIP1-like* was upregulated by Zn deficiency and moderately down-regulated by Zn excess, while *NtZIP11* was upregulated by Zn excess. Second, *NtZIP1-like* transcript was present in all vegetative organs at a moderate level and went up in young leaves of growing plants. In comparison, *NtZIP11* expression was more abundant in the shoots than in the roots and increased in the older leaves. Thus, although NtZIP1-like and NtZIP11 play a universal role in supplying cells with Zn under control conditions, they have different, more specific functions. NtZIP1-like is involved in supplying internal tissues of younger organs with Zn (primarily under deficiency), while NtZIP11 specifically participates in the uptake of Zn excess in plants exposed to its high, toxic concentrations, contributing to the accumulation of a high amount of this metal in older tobacco leaves (Papierniak et al. 2018; Kozak et al. 2019; Weremczuk et al. 2020).

Bioinformatic analysis showed the presence of ZDRE elements in the promoter of Zn-deficiency-inducible *NtZIP1-like*. In contrast, the promoter of *NtZIP11* upregulated by Zn excess contained a truncated ZDRE sequence in which the last two base pairs (bp) were missing. To determine promoter sequences responsible for the strong expression of *NtZIP11*, the activity of the short version of the *NtZIP11* promoter (containing 807 bp) was analyzed, (Supplementary File S1), and compared with the activity of the long promoter of 1922 bp, characterized by Weremczuk et al. (2020). In the transgenic plants stably expressing the *promNtZIP11*_*long*_*::GUS* construct, the activity of the long *NtZIP11* promoter under control conditions was found in the whole leaf blades, and increased upon exposure to elevated Zn. In the roots, however, Zn excess did not modify the tissue-specific *NtZIP11* expression; it was found in the internal tissues along the root length, but not in the apical and basal part (Weremczuk et al. 2020). To compare, the use of a short version of the *NtZIP11* promoter (807 bp) to drive *GUS* led to a complete loss of expression in the roots. In leaves, however, it remained at a very low level (Supplementary File S1). Interestingly, even with such low expression (compared with the long promoter of 1922 bp; Weremczuk et al. 2020), the ability to induce expression in the leaves in the presence of elevated Zn was retained. The short version did not contain two IDE2 elements (Iron Deficiency-responsive Element 2) responsible for the Fe-deficiency response (Kobayashi et al. 2003). Additional studies are needed to identify the sequences responsible for the Zn-dependent upregulation of *NtZIP11* in the leaves. Knowledge of this regulation could be used to modify the ability of tobacco to accumulate metals in leaves.

The next group of proteins involved in metal uptake belongs to the NRAMP family (Natural Resistance Associated Macrophage Proteins). They transport a range of metals, including iron (Fe), manganese (Mn), zinc, nickel (Ni) or cadmium (Nevo and Nelson 2006). In tobacco, NtNRAMP5 was implicated in Cd and Mn uptake (Tang et al. 2017). Two homologs were isolated from two cultivars differing in the efficiency of Cd accumulation in the shoots, Jinyjing and Komotini Basma (with lower and higher Cd accumulation capacity, respectively). The search for a genetic basis underlying this difference discovered that the distinct ability to store Mn and Cd was related to the transport activity of NtNRAMP5 plasma membrane-localized uptake proteins from both cultivars—named NtNRAMP5s and NtNRAMP5l. NtNRAMP5l (from cv. Komotini Basma) possesses full transport activity for Cd and Mn, while NtNRAMP5s (from cv. Jinyjing, less efficient metal accumulator) was unable to transport Mn and had a weak transport activity for Cd due to a mutation for early translation termination resulting in a truncated protein missing 104 amino acids in the C-terminus. Thus, allelic variation explained the differences in accumulation of Mn and Cd between tobacco cultivars, also indicating that NtNRAMP5 determined the efficiency of the influx of both metals. Very recently, another plasma membrane-localized transport protein NtNRAMP3 has been identified (Kozak et al. 2022). Interestingly, it possesses a quite unique capability to mediate uptake of up to seven substrates, including cadmium, iron, manganese, cobalt (Co), copper (Cu), nickel and, with the lowest efficiency, zinc. Extensive studies suggested its main function as maintaining metal cross-homeostasis preferentially in leaves. Another transporter from this family is *NtNRAMP1* (Sano et al. 2012). It encodes a plasma membrane protein involved in Fe uptake. Further research is needed to learn more about its substrates and to determine its function.

### Regulation of the root-to-shoot translocation of Zn and Cd

Metal ions taken up by roots are transported across the cortex towards the central cylinder, and then loaded into xylem vessels. This radial transport is accomplished through three pathways: (i) apoplastic (solutes diffuse in the apoplast); (ii) symplastic (via plasmodesmata); (iii) with the participation of influx and efflux transmembrane transporters (Barberon 2017; Ricachenevsky et al. 2018). However, at the endodermal layer, the apoplastic pathway is blocked due to the presence of Casparian Strips, forcing onward symplastic transport of ions to reach the central vasculature. Once in the xylem, metals are transferred in the transpiration stream to the shoots. The effectiveness of metal root-to-shoot translocation depends on two key processes: (i) the size of the metal pool available for xylem loading (which depends on the root capacity to retain metals, regulated by the coordinated influx and efflux processes, with the key contribution of vacuoles in sequestration); (ii) efficiency of loading metals into the xylem vessels (it depends on efflux transporters localized at the plasma membrane of the pericycle and xylem parenchyma cells) (Palmgren et al. 2008; Ricachenevsky et al. 2018).

Tobacco is known for its high efficiency in the translocation of Cd and Zn from the roots to the aerial plant parts (Wagner et al. 1986; Angelova et al. 2004; Jarup and Akesson 2009). However, the molecular mechanisms underlying this phenomenon has so far only been partially explored.

#### Zn and Cd retention in roots as a factor in efficient translocation to shoots

The pool of metals available in the roots for xylem loading depends on a range of complex molecular mechanisms controlling the effectiveness of their sequestration in cellular compartments, primarily in the cortex. Vacuoles are the major storage sites for metals. Their increased capacity to accumulate metals limits the efficiency of translocation to shoots by reducing the amount available for loading into the xylem (Palmgren et al. 2008). It has been reported that loss-of-function of transporters involved in Zn or Cd efflux into the vacuoles resulted in higher metal translocation to shoots, which was shown for example in *Arabidopsis thaliana* for *AtMTP3* (Metal Tolerance Protein Family) or in *Oryza sativa* for *OsHMA3* (Heavy Metal Transporting P1B-ATPase Family) (Arrivault et al. 2006; Miyadate et al. 2011). In tobacco, contribution of *MTPs* or *HMA3* to the regulation of the effectiveness of metal translocation has not been studied so far. Only Bazihizina et al. (2014) showed that exposition of tobacco to Zn excess (250 µM) enhanced the retention of this metal in root vacuoles. This was accompanied by a dose-dependent increase of *NtMTP1* expression, which confirms the possible involvement of this gene in zinc sequestration. Further detailed research is necessary to define its role in metal root/shoot distribution.

Recent studies have pointed to the involvement of the ZIP family gene, *NtZIP4A/B*, in the regulation of Zn and Cd translocation to tobacco shoots (Maślińska-Gromadka et al. 2021). Lowering the transcript level in the *NtZIP4A/B* RNAi plants by around 40% resulted in decreased translocation of both Zn and Cd, but, interestingly, this effect depended on a combination of the concentrations of both metals. Thus, a reduction of Zn translocation was detected at 1 µM Zn + 1 µM Cd (and not at 1 µM Zn + 0 µM Cd), while decreased Cd translocation at 0 µM Zn + 0.25 µM Cd (and not at 1 µM Zn + 1 µM Cd). Decreased translocation of Zn at 1 µM Zn + 1 µM Cd was associated with more metal retained by the middle and basal root parts, and with alteration of Zn localization (examined by Zn indicator Zinpyr1) across these root segments, namely by more efficient accumulation in the subepidermal cortex cells and on the border between the cortex and the stele, where endodermis is present. It was proposed that NtZIP4A/B is likely to contribute to the regulation of the efficiency of the symplastic radial transport of both Zn and Cd towards xylem, primarily in the middle and basal root part; its suppression increases the metal pool in the apoplast and limits radial transport via the endodermal barrier causing a reduction in the amount of metal available for loading into the xylem.

More detailed studies identified a range of mutual concentrations of Zn and Cd which modified the transfer rate of both metals to shoots (Barabasz et al. 2016; Palusińska et al. 2020). Importantly, the Zn/Cd status-dependent efficiency of root-to-shoot translocation of Zn and Cd was accompanied by modulation of the accumulation of both metals specifically in the root sectors (the apical, middle and basal). At Zn deficiency, metal concentrations in the root parts were similar. In contrast, with increasing levels of zinc and cadmium in the medium, the middle and basal parts became a sink for their excess, probably protecting the meristematic apical part from toxicity. Furthermore, for each combination of Zn/Cd concentrations (low–medium–high), different *ZIP* genes with modified expression were identified, suggesting distinct molecular mechanisms that contribute to the Zn/Cd status-dependent efficiency of their translocation to shoots. They have been categorized into four groups. The first includes genes with the highest expression in the apical part (*NtZIP2*, *NtZIP5A/B, NtIRT1, NtIRT1-like*), the second—those with the highest expression in the basal part (*NtZIP1-like, NtZIP8*), the third—*NtZIP4A/B* with the same level in all parts, and the fourth group—genes with distinct expression in the apical, middle and the basal root part (*NtZIP5-like, NtZIP11*) (Palusińska et al. 2020). Importantly, their expression was altered specifically in the root parts in a Zn/Cd status-dependent manner. For example, adding 0.25 µM Cd to the Zn-deficient medium decreased the expression of *NtIRT1* in the apical part, *NtZIP5-like* in the middle part, and *NtZIP5A/B* in all three root parts. Other changes were found when the Zn concentration increased, and also upon adding 1 µM Cd to the reference medium. In all cases, modulations of the expression profiles were also root part-specific. These results indicate that the Zn/Cd status-dependent distribution of metals between the root parts, in conjunction with the specific expression of metal homeostasis genes, are important factors determining the efficiency of Zn and Cd translocation to shoots at changing supplies of both metals. At present, we are far from understanding the mechanisms involved in the regulation of this complex phenomenon. The presented studies provide a range of candidate *ZIP* genes for further analysis aiming at elucidation of their contribution. Of course, not only *ZIP* genes are involved, therefore, identification of other metal transporters acting in concert will reveal all components of the homeostatic network responsible for the regulation of the Zn/Cd status-dependent root-to-shoot translocation of both metals.

#### Contribution of xylem loading to regulate Zn and Cd translocation to the shoot

Thus far, in numerous plant species (including hyperaccumulators) the best characterized key proteins regulating the efficiency of loading Zn and Cd into xylem vessels (and consequently translocation to shoots) are HMA4 and HMA2 (Mills et al. 2005; Hermand et al. 2014). They both were identified as Zn and Cd export proteins localized in the plasma membrane. Being expressed in the roots primarily in the xylem parenchyma and pericycle, they load both metals into xylem vessels, which determines the effectiveness of translocation. In tobacco, two orthologs of *A. thaliana HMA2* and *HMA4* were cloned and named *NtHMAα* and *NtHMAβ* (Hermand et al. 2014), later denominated as *NtHMA4.1* and *NtHMA4.2,* which share 97% identity within their coding region (Liedschulte et al. 2017). Both genes were expressed in the vascular tissue primarily in younger roots, while in the leaves it was only at a very low level. Studies performed on different tobacco cultivars confirmed their importance for Zn and Cd translocation to shoots. Silencing *NtHMA4.1* and *NtHMA4.2* (RNAi plants and double knockout mutants) impaired translocation to shoots of both metals, but also led to Zn deficiency symptoms within the shoots, thus, it was concluded that they are redundant for that function. A more detailed analysis of the consequences of *NtHMA4.1/4.2* silencing for plant performance showed various pleiotropic effects, indicating deregulation of the nutrient balance. Among others, changes were found in the uptake, distribution within organs, and accumulation pattern of many macroelements, e.g., phosphorus (P), nitrogen (N), iron (Fe), manganese (Mn), and copper (Cu). This pointed to another important function of HMA4, namely contribution to the regulation of Zn homeostasis and the balance between the micro- and macroelements in a plant body. Genes with altered expression accompanying the observed ionomic changes were also identified in tobacco (Liedschulte et al. 2021), providing excellent candidates for further investigation of how the complex homeostasis network works to keep the nutrient balance at Zn deficiency (here generated by blocking Zn transfer to the shoots).

### Mechanism of metal accumulation in leaves

Leaves are the major organs involved in the accumulation of metals. Recent studies showed that in tobacco exposed to elevated zinc concentrations, its large amounts were not accumulated evenly in the mesophyll tissue, but in the groups of palisade parenchyma cells called “Zn accumulating cells”, which enables non-accumulating neighbouring ones to fulfil their physiological functions. This physiological feature has been recognized as a protective mechanism for the whole organ against the toxicity of excess metals. With time, groups of “Zn accumulating cells” undergo programmed cell death (PCD) and form lesions, however, zinc remains within these areas due to lignified cell walls (Siemianowski et al. 2013; Weremczuk et al. 2017).

In a search for candidate genes potentially responsible for the unique capability of tobacco to accumulate a high amount of Zn in the shoots, the expression level of bioinformatically identified genes was assessed in the leaves after exposure to 200 µM Zn. Seven genes with strongly altered expression (as compared with the control conditions) were identified. These include *NtZIP1-like, NtZIP4, NtZIP11-like, NtNRAMP3-like, NtMTP2-X1/X2*, *NtMRP10-like* and *NtMRP14-like* (from the Multidrug Resistance-Associated Proteins Family) (Papierniak et al. 2018). Subsequent research indicated the involvement of *NtZIP1-like* and *NtZIP11* in Zn accumulation in leaf mesophyll cells and hypothesized that the regulation of their uptake function could be linked to wall-associated kinases (WAKs) NtWAK2/NtWAK4-dependent signalling pathways, likely involved in detecting the zinc status in leaves (Weremczuk et al. 2020). It has been suggested that the cell-specific loading of zinc into the “Zn accumulating mesophyll cells” is probably related to its level in the apoplast, which, after exceeding a threshold concentration generates the rhamnogalacturonan I (RG I) fraction of pectins. The obtained results provided indirect evidence based on which the hypothesis indicating the major players was put forward. It was proposed that the RG I fraction of pectins might bind to the extracellular domain of NtWAK2P-s, NtWAK4P-4 and/or NtWAK4P-2. As a result, a signal of high Zn is transmitted into the cytoplasm and to the nucleus and contributes to the regulation of the expression of *NtZIP1-like* and *NtZIP11* uptake transporters, which contribute to loading of Zn excess to the groups of “Zn-accumulating cells”.

Similarly, uneven expression of *NtZIP5B* was found in the mesophyll of the leaves from tobacco stably transformed with the *promNtZIP5B::GUS* construct. The GUS activity was detected in the groups of palisade parenchyma cells (Palusińska et al. 2020). However, given that *NtZIP5A/B* is upregulated by Zn limiting conditions (no expression at the control medium), such a pattern of expression is not likely to be related to the accumulation of excess Zn, but rather to the cell-specific supply of micronutrients under their low availability. It could be hypothesized that also under deficiency conditions, only groups of cells receive optimal Zn supply (at the expense of neighbouring ones), which in turn can be considered a protection mechanism of the leaf as a whole against Zn deficiency, as it enables some of the cells to perform photosynthetic functions.

Transporters belonging to the MTP family are considered key proteins in the regulation of metal accumulation as they are involved in the sequestration of metals in the vacuoles (Ricachenevsky et al. 2013). Two genes, *NtMTP1a/b* and *NgMTP1* were isolated from *N. tabacum* and *N. glauca,* respectively. Yeast complementation studies identified Zn and Co as substrates for both transporters. The encoded proteins were shown to be located in the tonoplast, suggesting their contribution to vacuolar sequestration and, consequently, a role in the reduction of metal-induced toxicity (Shingu et al. 2005). Similarly, the participation of *NtMTP1* and *NtMTP4*, and also *NtNRAMP1* in the accumulation of Zn and Cd in tobacco leaves was also indicated by Vera-Estrella et al. (2017).

Another gene from the MTP family, *NtMTP2*, was first identified as upregulated in the leaves by Zn excess (200 µM Zn) (Papierniak et al. 2018). However, further studies showed that it encodes the tonoplast-localized transporter involved in sequestration of Co and Ni, but not Zn, and its expression pattern suggested a role as a housekeeping gene (Papierniak-Wygladala et al. 2020). Noteworthy, the activity of the *NtMTP2* promoter (analyzed using a GUS-reporter protein) in the palisade parenchyma cells changed after administration of 200 µM Zn from uniform (under control conditions) to localized in groups of cells, although Zn is not a substrate for this protein. Such a cell-specific expression pattern of *NtMTP2* in the tobacco leaves mimics the pattern of *NtZIP1-like* and *NtZIP11* expression, genes that are known to be involved in Zn accumulation in the groups of “Zn-accumulating cells” (Weremczuk et al. 2020).

A possible explanation is that NtMTP2 is also involved in the regulation of metal cross-homeostasis. Cells with high *NtMTP2* promoter activity may be just "Zn accumulating cells" in which high concentrations of this metal impaired cross-homeostasis. A possible explanation is that cells with high *NtMTP2* promoter activity may be just "Zn accumulating cells", in which NtMTP2 is involved in the regulation of disturbed metal cross-homeostasis due to exposure to Zn excess.

Bioinformatics and subsequent RT-qPCR expression analysis provided a very good source of *MTP* genes for further research on their contribution to the accumulation of metals in tobacco (Liu et al. 2019). A total of 26 *NtMTPs* were identified and designated as *NtMTP1.1* to *NtMTP12.2*. The most important genes, considering potential involvement in the regulation of Zn and Cd transport in tobacco, were *NtMTP1.1*, upregulated by Cd, and *NtMTP6.1*, *NtMTP8.4,* and *NtMTP11.1*, upregulated by both Zn and Cd. There are also a few others with increased expression in the presence of Cd, such as *NtMTP1.2, NtMTP8, NtMTP9.1/9.2, NtMTP10.3/10.4*, and *NtMTP11.2*.

The role of NRAMP transporters in metal accumulation is different from that of MTP proteins. They participate, depending on the subcellular localization, in the opposite processes—uptake or redistribution of metals from intracellular stores. In tobacco, this group of proteins is very poorly recognized. Two metal uptake transporters NtNRAMP1 and NtNRAMP5, localized in the plasma membrane, were already described in chapter 2.1 (Zn and Cd uptake).

It is also worth noting that trichomes have an important function in the accumulation of metals in tobacco leaves. Recently, it was shown that long glandular trichomes are very active in the accumulation of Cd, which is followed by the excretion of this metal. Two genes (*NtHMA2* and *NtZIP4*) were strongly upregulated by Cd in these structures, and NtHMA2*,* as a Cd export protein, has been assigned a role in metal excretion from glandular trichomes (Zhang et al. 2021).

The role of the YSL (Yellow Stripe-Like) proteins, which mediate the transport of metals in the form of chelates, has also been indicated as contributing to the efficient accumulation of Zn and Cd in the shoots/leaves of tobacco (Hodoshima et al. 2007; Huang et al. 2021; Zhang et al. 2021). However, apart from suggesting the possibility of participation in the regulation of these processes, more specific data is not available yet. It seems that the encoded proteins may be one of the key elements distinguishing tobacco from other species that are less effective in the high accumulation of both metals in aerial parts.

## Tobacco in phytoremediation

Phytoremediation is a plant-based technology that enables the removal of contaminants (e.g., heavy metals, organic compounds) from the soil, water and air (Cunningham and Berti 1993). Thanks to such treatment, the concentration of pollutants in the environment and their toxicity are reduced (Greipsson 2011). There is a variety of phytoremediation techniques: (i) phytoextraction, (ii) phytostabilization, (iii) phytodegradation, (iv) phytofiltration, (v) phytovolatilization, (vi) plant-assisted bioremediation (microbial), (vii) soil supplementation with Zn; (viii) removal of aerial contaminants, (ix) phytodesalination (Singh et al. 2003; Suresh and Ravishankar 2004; Ali et al. 2013). However, phytoremediation technology itself is not the focus of this review. More information can be found in many reviews on this topic (Eapen et al. 2005; Fulekar et al. 2009; Kotrba et al. 2009; Maestri and Marmiroli 2011; Sarwar et al. 2017; Shawai et al. 2017; Muthusaravanan et al. 2018; Yan et al. 2020; Ozyigit et al. 2021;).

### Improving phytoremediation efficiency by genetic modifications

Tobacco is a good candidate for phytoextraction. It has high biomass with well-developed root system, high productivity and growth rate. Furthermore, it can be easily cultivated (does not have high fertilizer requirements) and harvested. Tobacco plant species also have a wide geographic distribution (Daghan 2019). Importantly, however, it also has the ability, unique among plants, to transfer efficiently Cd and Zn from the roots to the shoots and accumulating them there at high concentrations (Wagner et al. 1986; Angelova et al. 2004; Doroszewska and Berbeć 2004; Lugon-Moulin et al. 2004; Willers et al. 2005; Jarup and Akesson 2009). Therefore, tobacco has frequently been used for phytoextraction of these metals from contaminated soil. In a recent review, the use of tobacco for phytoremediation and applied management strategies were described (Rehman et al. 2019).

While quite satisfactory results have been obtained in many cases, there is nevertheless a need to increase the efficiency of Zn and Cd phytoextraction, mainly to shorten the time needed to achieve a satisfactory reduction of the metal content in the soil. Consequently, attempts have been made to introduce metal homeostasis genes into tobacco.

The concept of genetic modification of plants for phytoremediation purposes has been discussed in various manuscripts: Eapen et al. 2005; Fulekar et al. 2009; Kotrba et al. 2009; Maestri and Marmiroli 2011; Sarwar et al. 2017; Shawai et al. 2017; Muthusaravanan et al. 2018; Yan et al. 2020; Ozyigit et al. 2021). Briefly, for that purpose, several strategies have been introduced. They include the use of genes from different categories encoding: (i) metal transporters, (ii) metal-binding peptides and proteins, such as phytochelatins (PCs) and metallothioneins (MTs), or (iii) enzymes enabling degradation, oxidation, volatilization or conversion to less toxic forms, which are involved not only in altering metal transport but also in enhancing biomass production, developing root growth or increasing growth rate (Fulekar et al. 2009). In most attempts, genes were heterologously expressed under the constitutive promoter CaMV 35S. The genes used for tobacco transformation to modify tolerance to Cd and Zn, root/shoot distribution and accumulation in leaves are summarized in Supplementary Table S2.

#### Overexpression of metal transporter genes in tobacco

Metal transporters play an important role in the transfer of metal ions or metal complexes through biological membranes (plasma membrane and organellar membranes). It was postulated that improved Cd and/or Zn accumulation in the shoots/leaves of tobacco might be achieved by introducing genes encoding metal transporters from CAX (Cation EXchangers), HMA, MTP, MRP, YSL or ZIP families (Guerinot 2000; Axelsen and Palmgren 2001; Klein et al. 2006; Curie et al. 2009; Ricachenevsky et al. 2013; Pittman and Hirschi 2016).

Unfortunately, only two attempts to increase Cd and/or Zn accumulation in tobacco leaves by introducing genes encoding metal transporters were successful. Accordingly, these transgenic plants might be potentially applied in phytoextraction, however, more detailed studies should be performed.

In tobacco, heterologous expression of *OsMTP1* from *Oryza sativa* resulted in increased cadmium accumulation in both roots and shoots, while expression of *BjYSL7* from *Brassica juncea* caused higher Cd content only in the above-ground organs. Both modifications minimized the inhibitory effect of Cd on plant growth (Wang et al. 2013; Das et al. 2016). Interestingly, introducing *BjYSL7* into tobacco ensured longer roots with superior root hairs (Wang et al. 2013). Accordingly, overexpressing this gene in plants might be useful in increasing the ability of roots to penetrate the soil and remove metals from its deeper layers. In tobacco transformed with *OsMTP1*, the vacuolar thiol content was higher, which suggests that the presence of these chelating compounds might contribute to improving plants’ tolerance to Cd (Das et al. 2016).

The vast majority of genetic modifications aiming at enhancing Cd/Zn accumulation in tobacco shoots/leaves were not fully successful. The concentration of these metals in transgenic plants increased, but only in the roots. Nevertheless, reducing the rate of metal transport to the shoots might be useful in the tobacco industry. Preventing Cd translocation to leaves results in a lower Cd content in above-ground organs and, consequently, minimizes the negative influence of this metal on smokers’ health.

Tobacco plants expressing *AtCAX2* and *AtCAX4* from *A. thaliana* accumulated more Cd and Zn primarily in the roots. However, they were also more tolerant to the excess of both metals. While cultivated in a medium with high concentrations of Cd, their biomass was higher than in non-transformed plants. Moreover, in plants expressing *AtCAX2* increased Cd transport to the vesicles has been demonstrated (Hirschi et al. 2000; Korenkov et al. 2007). It was concluded that enhanced retention of cadmium excess in the root vesicles prevented its further transport to the above-ground organs.

Introducing transporters such as *AtMRP7* from *A. thaliana* or *ArsC* from *Escherichia coli* increased tobacco transformants’ tolerance to Cd and the accumulation pattern (Dhankher et al. 2003; Wojas et al. 2009). Plants overexpressing the bacterial gene *ArsC* could grow on a broad range of high Cd concentrations (up to 100 µM Cd), had a more vigorous phenotype than controls, and accumulated up to 50% more Cd in the shoots (Dhankher et al. 2003). On the other hand, expression of *AtMRP7* in tobacco resulted in more efficient retention of Cd in the roots, accompanied by enhanced sequestration of cadmium in the vacuoles (Wojas et al. 2009).

To increase the efficiency of the root-to-shoot translocation of Zn and Cd, *AtHMA4* from *Arabidopsis thaliana*, encoding a protein involved in loading Zn and Cd in root xylem (Mills et al. 2003; 2005) was extopically expressed in tobacco (Siemianowski et al. 2011). Three versions of *AtHMA4* were used; a complete sequence (AtHMA4-F), a truncated version lacking the C-terminal region (AtHMA4-trunc), and only the C-terminal region (AtHMA4-C). The C-terminal (key for protein activity) harbours putative heavy-metal-binding motifs, including thirteen Cys pairs and an eleven His stretch. Therefore, it was thought that in transgenic plants the C-terminal sequence might contribute to enhanced binding of Zn and Cd in the cytoplasm, which might result in higher accumulation and increased tolerance to both metals. Plants expressing these three constructs exhibited differences in response to high concentrations of Cd and Zn. Accumulation of Cd, as well as Zn, was lowered in tobacco transformed with both *AtHMA4-F* and *AtHMA4-trunc*, but not in *AtHMA4-C* plants. Overexpression of *AtHMA4-C* in tobacco caused up to a 4-fold increase in Cd and Zn accumulation in the roots and shoots (Siemianowski et al. 2011). It is worth emphasizing, that overexpression of *HMA4* sequences resulted in modifications in the translocation and accumulation of both metals, however, unexpectedly the pattern of changes depended on the Zn/Cd status in the medium. For example, in the presence of 0.5 μM Zn, accumulation of the metal was not modified in the shoots of *AtHMA4* expressing tobacco, while at 10 μM Zn it was higher as compared with non-transgenic plants (Siemianowski et al. 2011). Similar effects were seen after overexpression of two other genes in tobacco: *AhHMA4* from *A. halleri* and *HvHMA2* from *Hordeum vulgare* (Barabasz et al. 2010, 2013). It was concluded that the interplay between the transgene and the tobacco endogenous genes involved in the regulation of metal homeostasis contributed to the detected modifications. These issues have been extensively discussed in the review by Antosiewicz et al. (2014).

Concluding, most of the presented attempts to engineer tobacco with enhanced tolerance to Cd and/or Zn were accompanied by an increase in biomass. These traits together with more effective translocation, acquired by overexpression of certain genes encoding metal transporters, contribute to increased metal accumulation in leaves. Their shoot biomass could be easily harvested from the polluted area together with the stored metals.

#### Overexpression of genes encoding metal chelators

The metal tolerance and the ability to accumulate also depend on cysteine-rich metal-binding chelating molecules such as phytochelatins or metallothioneins (Hassinen et al. 2010; Merlos et al. 2014). Their amount in plants increases at exposure to metal stress. PCs bind metal ions and form complexes subsequently transported to vacuoles (Merlos et al. 2014; Goldsbrough 2020). PCs are small peptides synthesized enzymatically. To increase their concentration in plant tissues, genes encoding enzymes of the PC biosynthesis pathway were overexpressed. Metallothioneins bind metals also through the thiol groups of cysteine residues, however, inside the cells they are localised in the cytosol and do not seem to be transported into the vacuoles (Zhigang et al. 2006; Hassinen et al. 2010).

Overexpression of PCs in tobacco induced a variety of changes in Cd uptake and translocation. Only one out of three attempts increased the Cd content in the above-ground organs. Plants expressing *TaPCS1* from *Triticum aestivum* had enhanced tolerance to cadmium and accumulated 3.3-fold higher concentrations of Cd in the shoot. Unusually, the target organism was *N. glauca* R. Graham known as shrub tobacco (Gisbert et al. 2003; Martínez et al. 2006). Introducing into tobacco other genes encoding phytochelatin synthase such as *AtPCS1* from *A. thaliana* did not increase Cd root-to-shoot translocation factor (Pomponi et al. 2006; Zanella et al. 2016). Its accumulation was higher both in the roots and shoots, and was accompanied by enhanced synthesis of PCs and by higher Cd concentrations in the vacuoles (to a higher extent in underground organs) (Pomponi et al. 2006; Zanella et al. 2016).

The result of overexpression in tobacco of *PCS* gene also depended on the species of origin. Contrary to expectations, introduction of *AtPCS1* (from *A. thaliana*) into tobacco enhanced its sensitivity to Cd, which was accompanied by substantial increase in PCs and γ-glutamylcysteine concentrations and also by glutathione depletion. In contrast, expression of *CePCS* from *Caenorhabditis elegans* improved tolerance of tobacco to Cd, and transformants exhibited only moderate changes in the accumulation of PCs and γ-glutamylcysteine (Wojas et al. 2008). The results suggested that introduction of genes from the PCs synthesis pathway induced different responses of transgenic plants to Cd excess due to functional differences between AtPCS1 and CePCS proteins, which originated from evolutionarily distant organisms.

Metallothioneins are the second group of metal complexing compounds. Many attempts, as expected, increased the Cd content in the shoots.

The overexpression of *SaMT2* from *Sedum alfredii* in tobacco enhanced its tolerance to both Cd and Zn, increased accumulation in the shoots and, to a lesser extent, in the roots. The presence of zinc and cadmium also activated other detoxifying mechanisms, decreasing H_2_O_2_ content and increasing the activity of antioxidant enzymes (Zhang et al. 2014). These results indicate that coping with Cd excess involves several processes that enable effective detoxification.

Higher tolerance to Cd was also engineered in tobacco by the expression of *PjMT1* and *PjMT2* from *Prosopis juliflora* (Balasundaram et al. 2014; Krystofova et al. 2012). Furthermore, overexpression of these genes, as well as *CUP1* from *Saccharomyces cerevisiae*, resulted in increased Cd accumulation in the shoots (Macek et al. 2002; Thomas et al. 2003; Pavlíková et al. 2004a, b; Krystofova et al. 2012; Balasundaram et al. 2014). On the other hand, overexpressing *SvMThis* from *Silene vulgaris* in tobacco increased Cd accumulation primarily in the roots and, to a lesser extent, in leaves. However, the level of tolerance to Cd was not altered (Gorinova et al. 2007).

Overexpression of *MT* genes may also induce unexpected effects. In a few studies, Cd accumulation in the shoots was lower in transgenic plants compared with controls. Introduction of *hMT-II* from *Homo sapiens* and *MT-I* from *Mus musculus* decreased the cadmium concentration in the above-ground organs, especially in the leaf lamina, by even more than 70% (de Borne et al. 1998; Yeargan et al. 1992; Elmayan and Tepfer 1994). Plants overexpressing *hMT-II* also had lowered root-to-shoot translocation—only 20% of absorbed Cd was transported further to the shoots of transgenic tobacco (Elmayan and Tepfer 1994). The pattern of modifying the distribution of Cd as a result of transformation also depended on the tobacco cultivar. Thus, the expression of *hMT-I* in the cultivar cv. KY 14 resulted in 5% higher Cd accumulation in the roots and 24% lower in the shoots, whereas in cv. Petit Havana, it induced cadmium accumulation in the roots, while its leaf content did not change significantly (Yeargan et al. 1992). Alterations in plants’ response to a high metal concentration between cultivars indicate that processes involved in the regulation of homeostasis might be drastically different.

Genetic manipulation in the glutathione (GSH) biosynthesis pathway also contributed to enhancing Cd accumulation in the above-ground organs. Transgenic tobacco plants overexpressing *OAS-TL* from *Spinacia oleracea* grown in a medium with high concentrations of Cd exhibited higher biomass and root length relative to wild-type plants. Enhanced tolerance to Cd was accompanied by increased shoot metal content (Kawashima et al., 2004).

Modification strategies can also be combined. In the study by Grispen et al. (2011) genes encoding AtHMA4 and/or AtMT2b were introduced into tobacco and double (*AtHMA4* + *AtMT2b*) or single (*AtHMA4* or *AtMT2b*) transformants were generated. Overexpression of either *AtHMA4* or *AtMT2b* did not change the phenotype. However, in tobacco expressing *AtHMA4* + *AtMT2b* tolerance to Cd as well as Cd/Zn translocation were enhanced, but the response to zinc was unchanged.

To summarize, the strategy involving overexpression of genes encoding metal-binding proteins such as PCs or MTs in tobacco not always contributes to increase in Zn/Cd accumulation in the above-ground parts. There are cases of increasing the usefulness of tobacco for Zn and Cd phytoextraction. On the other hand, unforeseen changes happen, and overexpression of *MTs* in tobacco might contribute to lowering the metal content in the shoot, reducing translocation and retaining metals in the root zone. Such results are likely generated as pleiotropic effects occurring in transgenic plants after introducing genes from other organisms, in response to misbalancing metal homeostasis. This topic was discussed in detail in Antosiewicz et al. (2014).

### Tobacco engineered for reducing Cd translocation to the shoot

Considering the high efficiency of Cd accumulation in tobacco leaves, even from moderately contaminated soil, from the perspective of tobacco industry there is a need to minimize metal concentration in these organs to lower the exposure of smokers to this toxic metal. On one hand, attempts were made to limit Cd accumulation by the use of agronomic treatments (such as soil additives that increase the level of Zn) (Sarwar et al., 2010, 2017; Kinay et al., 2021). Another approach is to genetically modify tobacco for that purpose. To achieve this, there are two major alternative methodological approaches: (i) overexpression of selected metal transport genes or expression under tissue- or cell-specific promoters; (ii) silencing selected genes. Here we present the attempts to reduce Cd accumulation in tobacco leaves by silencing target genes involved in the control of Cd root-to-shoot transloation by: (i) decreasing the efficiency of Cd xylem loading; (ii) reducing the amount of Cd available for xylem loading.

As part of the first attempt to reduce Cd xylem loading efficiency, genes encoding proteins from the HMA sub-family were silenced in tobacco. In one study by Hermand et al. (2014), an artificial micro-RNA (amiRNA) technique was applied to lower the transcript level of *NtHMAα* and/or *NtHMAβ*. The designed constructs were used to inactivate (i) *NtHMAα*, (ii) *NtHMAβ* or (iii) both transcripts at the same time. Complete silencing was not observed in any of the transgenic lines, although *NtHMAα/β* abundance was significantly lower. Another approach was used, in which tobacco lines harboring a mutation in the *NtHMAα/β* gene were identified by screening an EMS (ethylmethanesulfonate)-mutagenized mutant collection with the use of CE-SSCP (Capillary Electrophoresis-Single Strand Conformation Polymorphism). In each of the *NtHMA* genes, missense and nonsense mutations that altered the sequence within the third cytosolic loop of HMA were detected. All plants showed reduced ability to accumulate Cd in the shoots (mutants and transgenic plants in which one or both *NtHMA* genes were silenced), and exhibited higher tolerance to Cd excess compared with controls. Thus, the applied methodological approach was successful to generate tobacco plants with a reduced ability to accumulate Cd in the leaves. Nevertheless, silencing of the *NtHMAα* and/or *NtHMAβ* gene(s) also changed plant development and impaired seed germination due to Zn deficiency, indicating significantly changed metal homeostasis probably due to pleiotropic effects (Hermand et al. 2014).

In other studies (Liedschulte et al. 2017, 2021) silencing *HMA4.1* and/or *HMA4.2* with RNAi technology caused a significant decrease in Cd accumulation in the shoots and up to 90% reduction of the root-to-shoot translocation rate (by silencing both genes). Consequently, the cadmium content in the leaves of transgenic plants was lowered to less than 10%. Moreover, only a single functional *AtHMA4* allele (*AtHMA4.1* or *AtHMA4.2*) can maintain cadmium in transformants on a similar level as in control plants. However, all transgenic plants exhibited altered morphology (e.g., retarded growth, necrotic lesions, altered leaf morphology). Thus, to reduce Cd accumulation in the leaves without developmental impairment, it was proposed to combine a nonsense mutation in one of the *NtHMA4* homologs, which causes a complete loss of function, with a missense mutation in the other *NtHMA4* gene causing functional reduction. However, in plants expressing *HMA4* the reduced Cd content was accompanied by lowered zinc levels in leaves and by local Zn-deficiency responses in these organs. These indicate that in tobacco, altering HMA4-dependent Cd translocation to the shoots also affects the homeostasis of other metals. Interestingly, it was shown that transgenic lines in which *HMA4* was silenced accumulated 4- to 6- fold more phosphorus (P), iron, manganese, and copper compared with control plants. Similar changes were detected in a field experiment, but to a lower extent, in the double mutant. *HMA4*-impaired lines had altered mineral-dependent responses in both leaves and roots, which also caused changes in organ appearance (e.g., necroses on leaves). These results point to HMA4 involvement not only in the regulation of Cd and Zn transport, but indirectly also in the homeostasis of other mineral nutrients in the shoot (Liedschulte et al. 2021) .

In the second method of reducing Cd accumulation in leaves, in which the goal was to lower the pool of metal available for xylem loading in the roots (thus minimizing Cd translocation), a metal transporter from the HMA family, *OsHMA3* from *O. sativa*, was chosen as a candidate gene. The protein is localized to the tonoplast and mediates vacuolar sequestration of cadmium. Tobacco expressing *OsHMA3* under the control of a root-specific promoter from a tobacco root extension-like protein-coding gene (*NtREL1*) resulted in the root-specific retention of cadmium, consequently lower root-to-shoot translocation and decreased accumulation in the shoots was generated (Cai et al. 2019).

Concluding, one of the good strategies to reduce Cd translocation to the above-ground organs and hence leaf concentration, is silencing selected metal transport genes. Such modifications, however, also affect the homeostasis of other nutrients, which might result in undesired changes in plant growth, and, accordingly, reduce the effectiveness of the applied genetic modification. Therefore, one should consider manipulating the level of reduction in the expression of selected genes to minimize Cd translocation to the shoots on the one hand, and to maintain the optimal level of micronutrient transport on the other.

## Prospects and conclusion

**Fig. 2:**
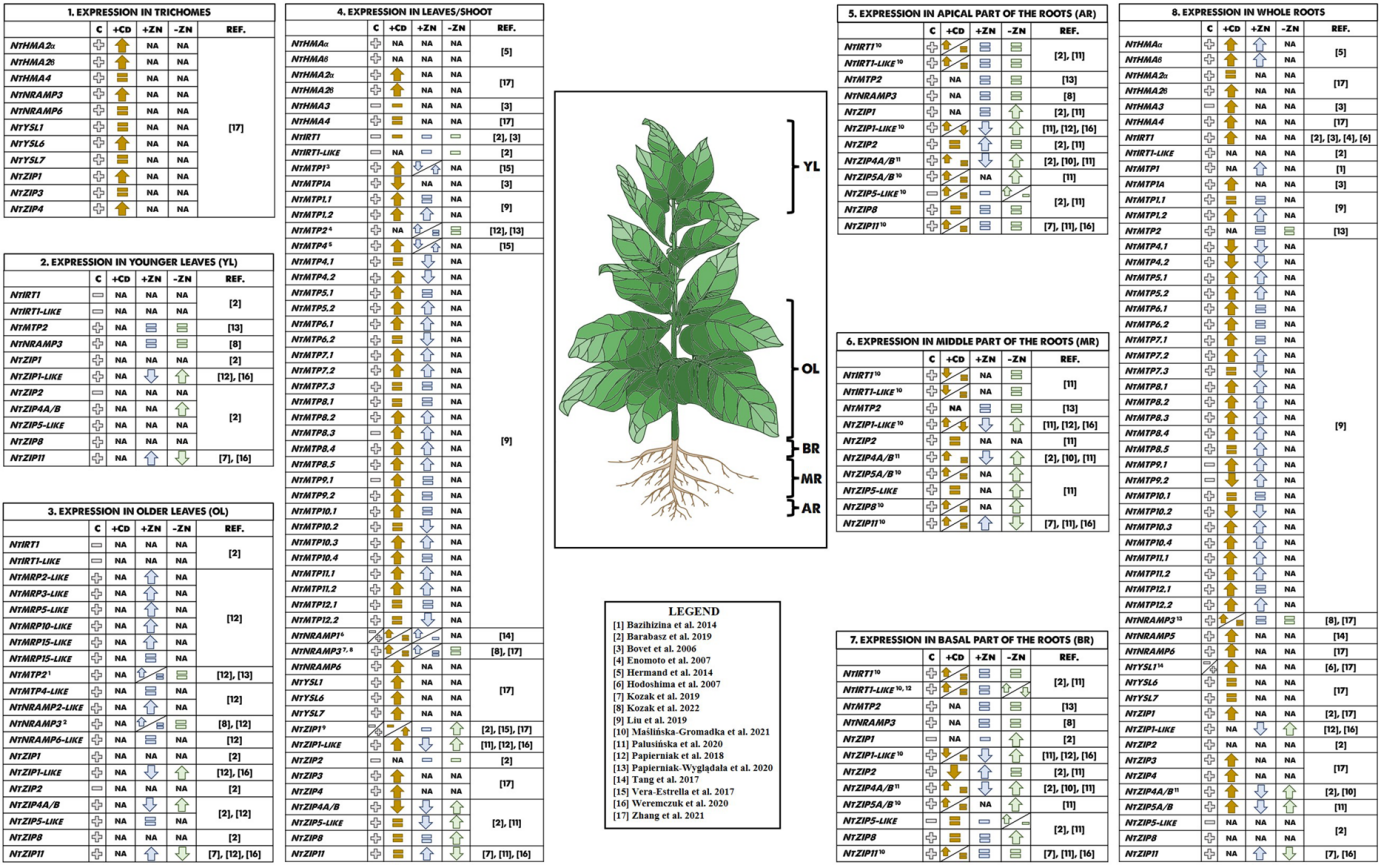
Regulation of Cd and Zn transport in tobacco. Genes involved in the regulation of Cd and Zn homeostasis in tobacco are sorted by their most characteristic expression sites. C control conditions, Cd cadmium excess,  + Zn zinc excess,  − Zn zinc deficiency, NA not analysed, up arrow upregulation, down arrow downregulation,  + transcript was detected,  − transcript was not detected,  = expression level was similar to control conditions, REF. references, YL young leaves, OL older leaves, AP apical part of the roots, MR midlle part of the roots, BR basal part of the roots. ^1^The expression in the older leaves was upregulated by Zn excess (RT-qPCR) (Papierniak et al. 2018), while changes in the expression level were not detected by GUS assay (Papierniak-Wygladala et al. 2020); ^2^The expression in the older leaves was induced by Zn excess (RT-qPCR) (Papierniak et al. 2018), while changes in the expression level were not detected by GUS assay (Kozak et al. 2022); ^3^The expression was downregulated by Zn excess in the leaves of juvenile plants, while in the leaves of adult plants expression was upregulated (Vera-Estrella et al. 2017); ^4^At control conditions: *NtNRAMP1* was expressed in the leaves of adult plants, but not in the leaves od juvenile plants; the expression was upregulated by Cd excess in the leaves of adult plants, while in the leaves of juvenile plants changes in the expression level were not detected; the expression was upregulated by Zn excess in the leaves of juvenile plants, while in the leaves of adult plants expression was not detected (Vera-Estrella et al. 2017); ^5^The expression was upregulated by Cd excess (Zhang et al. 2021), while changes in the expression level were not detected in (Kozak et al. 2022); ^6^The expression was upregulated by Zn excess (Kozak et al. 2022), while changes in the expression level were not detected in (Zhang et al. 2021); ^7^At control conditions: *NtZIP1* was expressed in leaves according to (Weremczuk et al. 2020), but not according to (Barabasz et al. 2019; Tang et al. 2017), at Cd excess: *NtZIP1* expression was not detected in the leaves of juvenile plants and upregulated in the leaves of adult plants, it was also upregulated according to (Weremczuk et al. 2020); ^8^The expression level changed due to time of the exposure to Cd; ^9^The expression level changed due to time of the exposure to Cd, the results differed according to the detection method (RT-qPCR or GUS assay); ^10^The expression level was upregulated according to (Palusińska et al. 2020), while expression was not detected according to (Barabasz et al. 2019); ^11^The expression level was upregulated by Cd excess according to (Zhang et al. 2021), while changes in the expression level were not detected according to (Kozak et al. 2022); ^12^At control conditions expression was detected according to (Zhang et al. 2021), but not according to (Hodoshima et al. 2007).

We are currently far from understanding the mechanisms of tobacco’s unusual efficiency of Cd and Zn root-to-shoot translocation. The genes known to date regulating metal homeostasis in tobacco are presented in the summarizing Fig. 2. Based on the available research results, it seems clear that one of the main problems is to determine the dependence of the efficiency of metal root-to-shoot translocation on nutrient composition. Due to the involvement of many genes, capturing key ones in regulating cross homeostasis is difficult, and will be a major challenge for future studies. Research on these complex processes is important not only to understand the regulation of tobacco's response to fluctuating mineral supply and to excess metals, but might also have wide practical application in genetic modification of tobacco and other species.

Many attempts have been made to genetically modify tobacco, depending on the needs—either to increase or to reduce the accumulation of cadmium in the leaves. However, they were only partially successful, and some have ended with different results than expected. One of the main reasons is the response of a host plant’s metal cross-homeostasis network to changes generated by the activity of a foreign protein that alters the status of metals in cells and tissues. These pleiotropic effects contribute to the generation of unforeseen changes (Antosiewicz et al. 2014). Future research should take this into account. Furthermore, identifying tissue- and cell-specific promoters and using them for engineering desired features could be of significant help.

### Supplementary Information

Below is the link to the electronic supplementary material.

Supplementary file1 (PDF 477 kb)— NtZIP11 promoter activity in tobacco

Supplementary file2 (DOCX 102 kb)—Expression profiles of tobacco genes

Supplementary file3 (DOCX 54 kb)—Genes used for genetic modifications of tobacco
