# Correction to: Tobacco as an efficient metal accumulator

**DOI:** 10.1007/s10534-023-00541-6

**Published:** 2023-09-22

**Authors:** Katarzyna Kozak, Danuta Maria Antosiewicz

**Affiliations:** Department of Plant Metal Homeostasis, Faculty of Biology, Institute of Experimental Plant Biology and Biotechnology, University of Warsaw, 1 Miecznikowa Str, 02-096 Warsaw, Poland

## Correction to: BioMetals (2022) 36:351–370 https://doi.org/10.1007/s10534-022-00431-3

In the above-mentioned publication, the reference to the original graphic of tobacco was not provided. The missing reference is: https://www.dreamstime.com/basic-cmyk-image163662662. The graphic was modified according to Royalty Free license.

