# Supplementary material for: Tobacco as an efficient metal accumulator
Source: Biometals. 2022 Sep 12;36(2):351–70. doi: 10.1007/s10534-022-00431-3 (PMC10082116; doi:10.1007/s10534-022-00431-3)
Supplement: Supplementary file 1 — Supplementary file1 (PDF 477 kb)— NtZIP11 promoter activity in tobacco [file 10534_2022_431_MOESM1_ESM.pdf]

Article title: **Tobacco as an efficient metal accumulator**

Journal name: **Biometals**

Author names: **Katarzyna Kozak and Danuta Maria Antosiewicz**

Affiliation: **University of Warsaw, Faculty of Biology, Institute of Experimental Plant Biology and Biotechnology, 1 Miecznikowa Str. 02-096 Warszawa, Poland**

E-mail Address of the Corresponding Author: **dma@biol.uw.edu.pl**

Supplementary File S1

Content:

A. **Identification, amplification and analysis of the *promNtZIP11*$_{short}$ sequence**
B. **Comparison of *cis*-acting regulatory elements of the *promNtZIP11*$_{short}$ and *promNtZIP11*$_{long}$ sequences (including 5' UTR of *NtZIP11*).**
C. **Tissue-specific expression of *NtZIP11* in *promNtZIP11*$_{long}$*::GUS* and *promNtZIP11*$_{short}$*::GUS* plants**

A. Identification, amplification and analysis of the *promNtZIP11*$_{short}$ sequence

In the previous study, *NtZIP11* promoter comprised of 1922 bp upstream from the START codon was described and used for determination of GUS-based *NtZIP11* spatial expression pattern. (Weremczuk et. al. 2020) Here, we provided a similar experimental approach, however with the use of shortened to 807 bp sequence of *NtZIP11* promoter named *promNtZIP11*$_{short}$ (see below). The aim of the study was to determine whether absence of several regulatory elements will influence the spatial expression pattern of *NtZIP11*.

Sequence of *promNtZIP11*$_{short}$:

5'UTR of *NtZIP11* (from the contig AWOK01S219909.1 position 7362…8168 – without ATG)

>promNtZIP11short
ATATACTTTACCGAATCATTCTTAAAAAGTTATCCAAAGAGTTGCAAAAGTTACAAAAAAATACTAAGTGGGTGT
TTGGCTAAGCTTATAAACCGGTCAAATTGATTTATAAGTAATTTTTGGCTTATTTATGCGTTTGGTAAAATTAAA
AGTGTTTATAAGTTAAGTGCTTATAAGTTAAAAGTCATAAGTTGGTCTCCTCCCACTTATCAAATTTCAGCTTAT
AAGCACTTTAGGTTTGACCAAAATATTTATTATTCTATCCCTAAAATAATTTTTTTTAAACAAAACTCTTCGTAT
ACCCAGTTCTTCAACTGTTTATTATTAATTTCAGTACTTTTATCCAAACACGTAACTTTTTATTTTTTAAATCAG
TTTCAGCATTTAAAAGTATTTTTCAGCACCTAATGCTTATCAGCTACTCTAAGCTAAACCAAACGGGTTCTAAAT
TACATGCTCTTGGGATGTGTATCATGTGACGAGTCATTAAAAACGTCAACCAATTTTCTTTTTTTTTTTCTCAAC
CAATTGTATTTATGGTAAATAGTAGTAGTACCATATATCAAGATTCACCAAAATGTCGACCAATTATATTTATGG
TGAGTAGTAGTACCGTACTTTCACATGTTCCATTTTTACGTCAAATACAAACAAAACTCTCCATTGCATTTGCAG
TACGCTGATTTTCTCTCTCTCATGTCTTAATCTCTTTGTCGTGCAGTACTACACCAAATTTAACCACTACTACAC
TATATAAAAACACACACACATCTCCAATCTTCC**CTCAATGATTTTCACTAAACTCCA****ATG**

ATG – START codon
ATATACTT – sequence of the forward primer and complementary sequence to the reverse primer

The procedure of promoter amplification and vector construction was the same as described in Weremczuk et. al. (2020). In brief, the *promNtZIP11*$_{short}$ sequence was amplified from genomic DNA with the use of appropriate primers (Table 1.) by PCR (Phusion HF polymerase; Thermo Scientific), subcloned to pENTR/D-TOPO and recombined

with pMDC163 to obtain the *pMDC163::promNtZIP11$_{short}$::GUS* construct. The correctiveness of the insert sequence was confirmed by sequencing.

Table 1. Primers used for amplification of *promNtZIP11$_{short}$* sequence.
* - primer reverse was the same for amplification of *promNtZIP11$_{short}$* or *promNtZIP11$_{long}$* sequences and its sequence was already published in Weremczuk et. al. (2020).

PRIMER NAME	PRIMER SEQUENCE	LENGTH OF AMPLICON
promNtZIP11short_F	5' CACCATATACTTTACCGAATCATTCTTA 3'	807 bp
promNtZIP11_R*	5' TGGAGTTTAGTGAAAATCATTGAGGGAA 3'	

To identify *cis*-acting elements within the *NtZIP11* promoter sequence we used the PlantCARE program (http://bioinformatics.psb.ugent.be/webtools/plantcare/html/). Results are given in Table 1, and in Figure 1.

Table 1. *Cis*-regulatory elements in the sequences of *promNtZIP11$_{short}$*.

***cis*-ACTING ELEMENT**	**SEQUENCE**	**POSITION IN THE PROMOTER SEQUENCE** ***promNtZIP11$_{short}$***	**REF.**
**A. METAL RESPONSIVE ELEMENTS**			
**ZDRE**	(A/G)TGTCGACA(T/C)	578	Assunção et. al. 2010
**IDE1**	CATGC	453	Kobayashi et. al. 2007
**MRE1**	TGC(A/G)(C/G)(A/C/G/T)C	671, 717	Li et. al. 2013, Qi et. al. 2007
**B. GENERAL RESPONSIVE ELEMENTS**			
TATA-box	ATATAA	752	Basehoar et. al. 2004
**CAAT-box**	(C)C(A)AAT	multi-position	Frangeul et. al. 2004
**C. PHYTOHORMONE RESPONSIVE ELEMENTS**			
CGTCA-motif	CGTCA	494, 639	Fink et. al. 1988
TGACG-motif	TGACG	477	Wang et. al. 2011
**D. LIGHT RESPONSIVE ELEMENTS**			
BOX4	ATTAAT	324	Hiratsuka et. al. 1997, Weisshaar et. al. 1991
BOX III	atCATTTTCACt	788	Willmitzer et. al. 1988
**E. ABIOTIC STRESS RESPONSIVE ELEMENTS**			
ARE	AAACCA	431	Paul & Ferl, 1991
MBS	CAACTG	312	Liu et. al. 1994

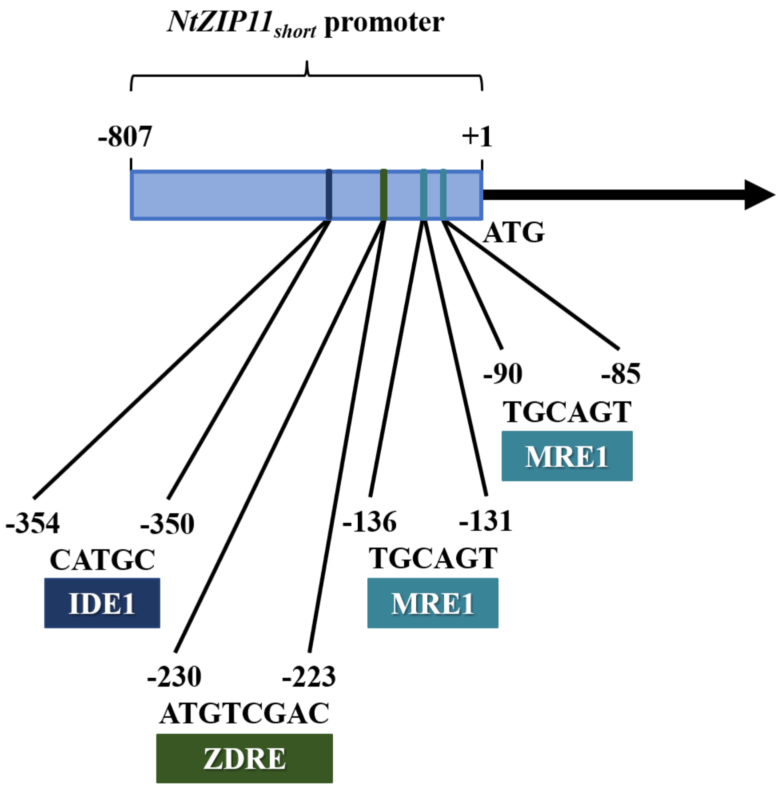

Fig. 1. Metal responsive regulatory elements in the sequence of *promNtZIP11*$_{short}$.

B. Comparison of *cis*-acting regulatory elements of the *promNtZIP11*$_{short}$ and *promNtZIP11*$_{long}$ sequences (including 5' UTR of *NtZIP11*)

Activity of the *promNtZIP11*$_{long}$ was characterized by Weremczuk et al. (2020). Due to its shortening, here it was shown that resulting *promNtZIP11*$_{short}$ sequence did not contain the following elements:

(1) IDE2 involved in Fe-deficiency response (Ogo et al. 2008);

(2) ABRE, TGA-box, TGA-element, CGTCA-motif, TGACG-motif involved in response to phytohormones (Fink et al. 1988; Shen and Ho 1995; Wang et al. 2011; Ahn et al. 2012; Zhu et al. 2014)

(3) ACT-motif, G-box, GATA-motif, I-box, LTR, TCCC-motif involved in light response (Lafyatis et al. 1991; Brown et al. 2001; Sibéril et al. 2001; Chawla et al. 2004; Natali et al. 2007; Mongkolsiriwatana et al. 2009).

C. Tissue-specific expression of *NtZIP11* in *promNtZIP11*$_{long}$*::GUS* and *promNtZIP11*$_{short}$*::GUS* plants

All plants used in this study (wild-type and transgenic) were based on the background of *Nicotiana tabacum* var. Xanthi. Seeds were obtained from the stock of the Institute of Biochemistry and Biophysics PAS (Warsaw, Poland; in 2002) and then propagated in the greenhouse of the University of Warsaw.

Plants cultivation and transformation were previously described in Weremczuk et. al. (2020). Applying the same approach, we generated *promNtZIP11*$_{short}$*::GUS* transgenic homozygous tobacco lines. Experiments were performed on eight independent homozygous lines.

Spatial expression pattern of *NtZIP11* in *promNtZIP11$_{short}$::GUS* plants was determined based on the methodology described in Weremczuk et. al. (2020). In brief, transgenic tobacco seeds were sterilized and germinated on Petri dishes with ¼ Knop's medium. After 3 weeks plants were transferred into hydroponics (aerated ¼ Knop's medium; 1.3 L pots) for three days and then subjected to (i) Zn deficiency (no Zn added to the medium) or (ii) zinc excess (20 µM $ZnSO_4$). In parallel, plants were grown at control conditions (¼ Knop's medium). Plant material (whole plants) was collected after 4 days and subjected to the GUS histochemical staining procedure described in Weremczuk et. al. (2020). In addition, transverse sections were made through the middle part of the roots. For this purpose, the root fragments were embedded in 3% agarose and cut (thickness: 130 µm) by Vibratome (Leica VT10000S, Heidelberg, Germany).

In the leaves of 4-week-old *promNtZIP11short*::GUS plants the NtZIP11short promoter activity was faint (Figure 2). The blue colour was present mainly in the veins, and with less intensity between them. Quantitatively, the promoter activity remained at similar level at control conditions and at Zn-deficiency (Fig. 2 a-b), and increased at enhanced zinc (20 µM; Figure 2c). Qualitatively, the NtZIP11 spatial expression pattern was similar at all applied treatments. In general, the activity of the *promNtZIP11short* was much weaker compared to that of the *promNtZIP11long* (Weremczuk et. al. 2020).

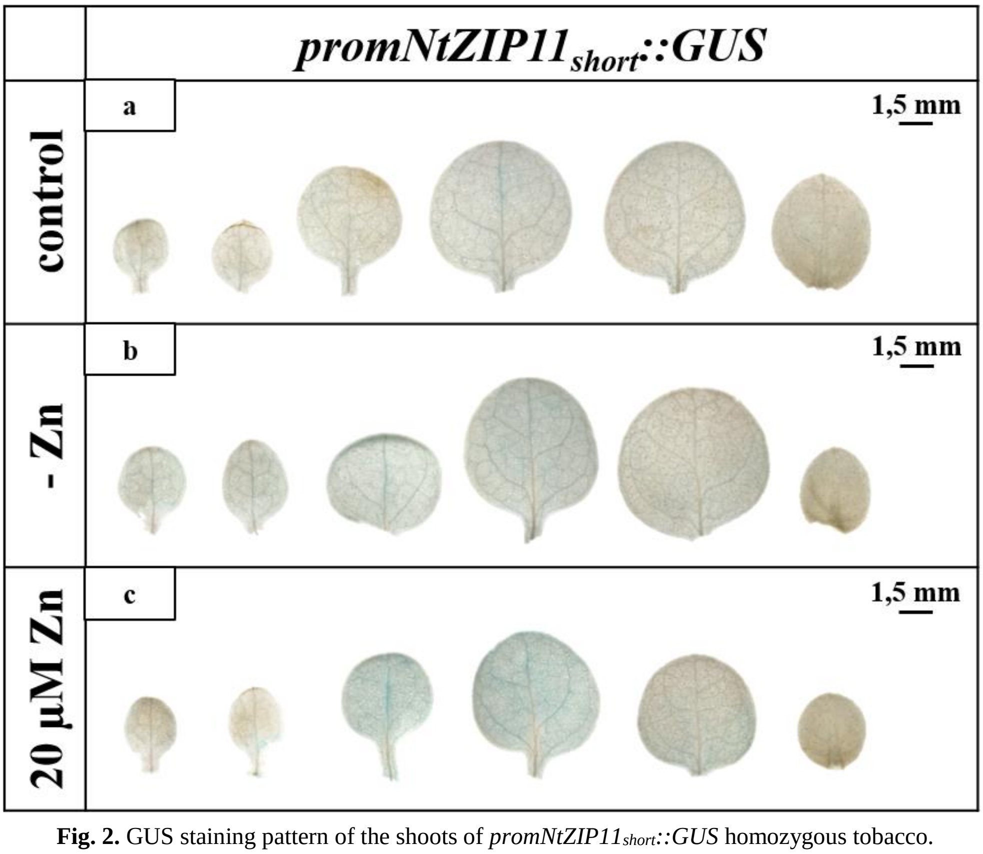

Fig. 2. GUS staining pattern of the shoots of *promNtZIP11$_{short}$::GUS* homozygous tobacco. Plants were grown in hydroponics (¼ Knop's medium) for 3.5 weeks and then were subjected to different zinc regimes: a. control conditions; b. zinc deficiency (no Zn was added to the medium); c. zinc excess (20 µM Zn) for 4 days.

No promoter activity was detected in the roots of *promNtZIP11$_{short}$::GUS* plants (Figure 3). In the previous study expression of *NtZIP11* in homozygous *promNtZIP11$_{long}$::GUS* tobacco was observed in the middle part of the main root and the lateral roots. The *NtZIP11* expression was downregulated by zinc deficiency, and upregulated by zinc excess (Weremczuk et. al. 2020). No staining was detected in wild-type plants (data not shown).

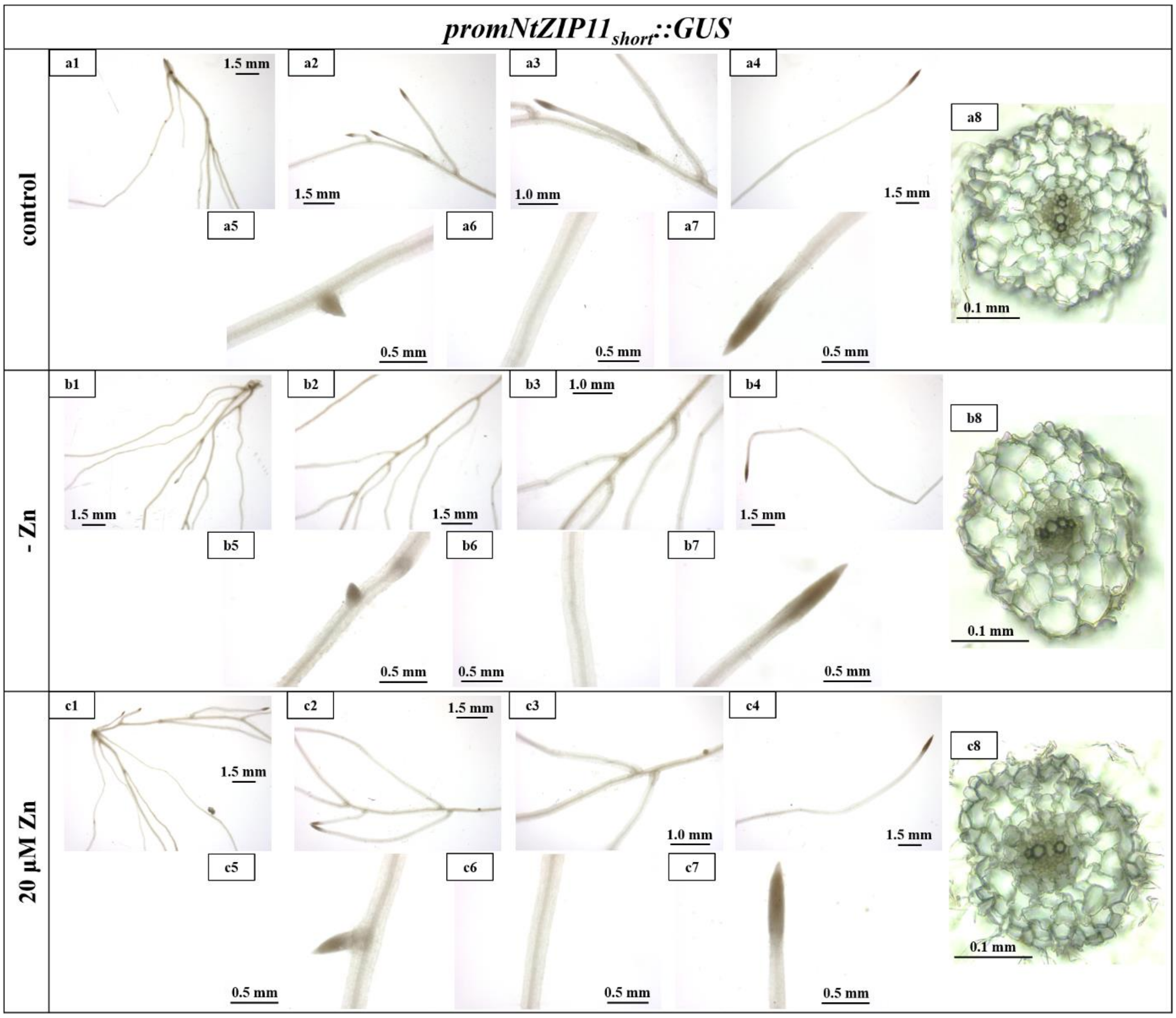

Fig. 3. GUS staining pattern in the roots of *promNtZIP11*$_{short}$*::GUS* homozygous tobacco. Plants were grown in hydroponics (¼ Knop's medium) for 3.5 weeks and then were subjected to different zinc regimes: a1-a8. control conditions; b1-b8. zinc deficiency (no Zn was added to the medium); c1-c8. zinc excess (20 µM Zn) for 4 days. Basal part of the root: a1, b1 and c1; middle parts of roots: a2-a3, a5-a6, b2-b3, b5-b6, c2-c3, c5-c6; apical part of the root: a4, a7, b4, b7, c4, c7; cross-sections of the middle part of the main root: a8, b8, c8.

References:

1. Ahn, Y.O., Kim, S.H., Lee, J., Kim, H., Lee, H.S., Kwak, S.S. 2012. Three Brassica rapa metallothionein genes are differentially regulated under various stress conditions. Mol. Biol. Rep. 39(3), 2059–2067
2. Assunção AG, Schat H, Aarts MG (2010) Regulation of the adaptation to zinc deficiency in plants. Plant Signal Behav 5(12):1553-1555. https://doi.org/10.4161/psb.5.12.13469
3. Basehoar AD, Zanton SJ, Pugh BF (2004) Identification and distinct regulation of yeast TATA box-containing genes. Cell 116(5):699-709. https://doi.org/10.1016/S0092-8674(04)00205-3
4. Brown, A., Dunn, M., Goddard, N., Hughes, M. 2001. Identification of a novel low-temperatureresponse element in the promoter of the barley (Hordeum vulgare L) gene blt101.1. Planta. 213(5), 77-780.
5. Chawla, R., DeMaso, D.A. 2004. Molecular expression of PsPIN1, a putative auxin efflux carrier gene from pea (Pisum sativum L.). Plant Growth Reg. 44(1),1-14.
6. Fink JS, Verhave M, Kasper S, Tsukada T, Mandel G, Goodman RH (1988) The CGTCA sequence motif is essential for biological activity of the vasoactive intestinal peptide gene cAMP-regulated enhancer. PNAS 85(18):6662-6666. https://doi.org/10.1073/pnas.85.18.6662
7. Frangeul L, Glaser P, Rusniok C, Buchrieser C, Duchaud E, Dehoux P, Kunst F (2004) CAAT-Box, contigs-assembly and annotation tool-box for genome sequencing projects. Bioinformatics 20(5):790-797. https://doi.org/10.1093/bioinformatics/btg490
8. Hiratsuka K, Chua NH (1997) Light regulated transcription in higher plants. J Plant Res 110(1):131-139. https://doi.org/10.1007/BF02506852
9. Kobayashi T, Ogo Y, Itai RN, Nakanishi H, Takahashi M, Mori S, Nishizawa NK (2007) The transcription factor IDEF1 regulates the response to and tolerance of iron deficiency in plants. PNAS 104(48):19150-19155. https://doi.org/10.1073/pnas.0707010104
10. Lafyatis, R., Denhez, F., Williams, T., Sporn, M., Roberts, A. 1991. Sequence specific protein binding to and activation of the TGF-β3 promoter through a repeated TCCC motif. Nucl. Acids Res. 19(23):6419–6425.
11. Li Y, Zhang Y, Shi D, Liu X, Qin J, Ge Q et al (2013) Spatial-temporal analysis of zinc homeostasis reveals the response mechanisms to acute zinc deficiency in Sorghum bicolor. New Phytol 200(4):1102-1115. https://doi.org/10.1111/nph.12434
12. Liu ZB, Ulmasov T, Shi X, Hagen G, Guilfoyle TJ (1994) Soybean GH3 promoter contains multiple auxin-inducible elements. Plant Cell 6(5):645-657. https://doi.org/10.1105/tpc.6.5.645
13. Mongkolsiriwatana, C., Pongtongkam, P., Peyachoknagul, S. 2009. In silico Promoter Analysis of Photoperiod-Responsive Genes Identified by DNA Microarray in Rice (Oryza sativa L.). Nat. Sci. 43,164 – 177
14. Natali, L., Giordani,T., Lercari, B., Maestrini, P., Cozza, R., Pangaro, T., Vernieri, P., Martinelli, F., Cavallini, A. 2007. Light induces expression of a dehydrin-encoding gene during seedling de-etiolation in sunflower (Helianthus annuus L.). J. Plant Physiol. 164(3), 263-273.
15. Ogo Y, Kobayashi T, Itai RN, Nakanishi H, Kakei Y, Takahashi M et al. (2008) A novel NAC transcription factor, IDEF2, that recognizes the iron deficiency-responsive element 2 regulates the genes involved in iron homeostasis in plants. J Biol Chem 283(19):13407-13417. https://doi.org/10.1074/jbc.M708732200

16. Paul AL, Ferl RJ (1991) In vivo footprinting reveals unique cis-elements and different modes of hypoxic induction in maize Adh1 and Adh2. Plant Cell 3(2):159-168. https://doi.org/10.1105/tpc.3.2.159
17. Qi X, Zhang Y, Chai T (2007) Characterization of a novel plant promoter specifically induced by heavy metal and identification of the promoter regions conferring heavy metal responsiveness. Plant Physiol 143(1):50-59. https://doi.org/10.1104/pp.106.080283
18. Shen, Q., Ho, T.H. 1995. Functional dissection of an abscisic acid (ABA)-inducible gene reveals two independent ABA-responsive complexes each containing a G-box and a novel cis-acting element. The Plant Cell. 7, 295-307.
19. Sibéril, Y., Doireau, P., Gantet, P. 2001. Plant bZIP G-box binding factors. Modular structure and activation mechanisms. Eur. J. Biochem. 268:5655–5666
20. Wang Y, Liu GJ, Yan XF, Wei ZG, Xu ZR (2011) MeJA-inducible expression of the heterologous JAZ2 promoter from Arabidopsis in Populus trichocarpa protoplasts. J Plant Dis Prot 118(2):69-74. https://doi.org/10.1007/BF03356384
21. Weisshaar B, Block A, Armstrong GA, Herrmann A, Schulze-Lefert P, Hahlbrock K (1991) Regulatory elements required for light-mediated expression of the Petroselinum crispum chalcone synthase gene. Proc Soc Exp Biol Med 45(6).
22. Weremczuk A, Papierniak A, Kozak K, Willats WG, Antosiewicz DM (2020) Contribution of NtZIP1-like, NtZIP11 and a WAK-pectin based mechanism to the formation of Zn-related lesions in tobacco leaves. Environ Exp Bot 176:104074. https://doi.org/10.1016/j.envexpbot.2020.104074
23. Willmitzer L (1988) The use of transgenic plants to study plant gene expression. Trends in Genet 4(1):13-18. https://doi.org/10.1016/0168-9525(88)90122-9
24. Zhu, Y., Wu, N., Song, W., Yin, G., Qin, Y., Yan, Y., Hu, Y. 2014. Soybean (Glycine max) expansin gene superfamily origins: segmental and tandem duplication events followed by divergent selection among subfamilies, BMC Plant Biol.14, 93
