# Supplementary material for: Tobacco as an efficient metal accumulator
Source: Biometals. 2022 Sep 12;36(2):351–70. doi: 10.1007/s10534-022-00431-3 (PMC10082116; doi:10.1007/s10534-022-00431-3)
Supplement: Supplementary file 2 — Supplementary file2 (DOCX 102 kb)—Expression profiles of tobacco genes [file 10534_2022_431_MOESM2_ESM.docx]

Article title: **Tobacco as an efficient metal accumulator**

Journal name: **Biometals**

Author names: **Katarzyna Kozak and Danuta Maria Antosiewicz**

Affiliation: **University of Warsaw, Faculty of Biology, Institute of Experimental Plant Biology and Biotechnology, 1 Miecznikowa Str. 02-096 Warszawa, Poland**

E-mail Address of the Corresponding Author: **dma@biol.uw.edu.pl**

**Supplementary Table S1**

**Supplementary Table S1.** Regulation of discussed tobacco genes by the deficiency or excess of selected metals.

FRO - ferric chelate reductase gene; HMA – genes encoding Heavy Metal Associated Proteins; MRP - genes encoding Multidrag Resistance Proteins; MTP - genes encoding Metal Tolerance Proteins; NAS – gene encoding Nicotianamine Synthase; NRAMP - genes encoding Natural Resistance-Associated Macrophage Proteins; YSL - genes encoding Yellow Stripe-Like Proteins; ZIP - genes encoding Zrt-/Irt-like Proteins; SB – substrates; SL – subcellular localization; NUC – nucleus; PM – plasma membrane; V – vacuole; R – roots; S/L – shoot/leaves; + transcript was present; - transcript was not detected; ↓ downregulation; ↑ upregulation; = control level; * expression depends on the plant age; ** detected in whole seedlings; *** expression depends on the root fragment, Cd concentration and time after treatment; **** the transcript was renamed from NtZIP1 to NtZIP5A according to Palusińska et al. 2020; empty boxes – expression was not determined.

| **GENE** | **METAL STATUS** | | | | | | | | **SB** | **SL** | **REF.** |
| --- | --- | --- | --- | --- | --- | --- | --- | --- | --- | --- | --- |
|  | **Control** | | **Zn** | | | | **Cd** | |  |  |  |
|  |  |  | **deficiency** | | **excess** | | **excess** | |  |  |  |
|  | **R** | **S/L** | **R** | **S/L** | **R** | **S/L** | **R** | **S/L** |  |  |  |
| *NtIRT1* | + | - | = | - | = | - |  |  |  |  | [2] |
| *NtIRT1* |  |  | ↑ |  |  |  | ↓/=*** | - |  |  | [13] |
| *NtIRT1* | - | - |  |  |  |  | ↑ | - |  |  | [4] |
| *NtIRT1* | + | + |  |  |  |  |  |  |  |  | [5] |
| *NtIRT1* | - |  |  |  |  |  | ↑ |  |  |  | [7] |
| *NtIRT1*** | +** | +** |  |  |  |  | ↑** | ↑** |  |  | [21] |
| *NtIRT1-like* | + | - | ↑ | - | = | - |  |  |  |  | [2] |
| *NtIRT1-like* |  |  | ↑ |  |  |  | ↓/=*** | - |  |  | [13] |
| *NtZIP1* | + | -/+* |  |  | =/↓* | - | ↑ | -/ ↑* |  |  | [20] |
| *NtZIP1* |  |  |  |  |  |  | ↑ | ↑ |  |  | [22] |
| *NtZIP1-like* | + | + | ↑ | ↑ | ↓ | ↓ |  |  | Zn | PM | [14] |
| *NtZIP1-like* |  |  | ↑ |  |  |  | ↓/=/↑*** | ↑ |  |  | [13] |
| *NtZIP2* | + | - | = | - | ↑ | - |  |  |  |  | [2] |
| *NtZIP2* |  |  |  |  |  |  | =/↓*** | - |  |  | [13] |
| *NtZIP3* |  |  |  |  |  |  | ↑ | ↑ |  |  | [22] |
| *NtZIP4* |  |  |  |  |  |  | ↑ | ↑ |  |  |  |
| *NtZIP4A* | + | + | ↑ | ↑ | ↓ | ↓ | = | = |  |  | [2] |
| *NtZIP4A* |  |  | ↑ |  |  |  | =/↑*** | ↓ |  |  | [13] |
| *NtZIP4B* | + | + | ↑ | ↑ | ↓ | ↓ | = | = | Zn, Cd | PM | [2] |
| *NtZIP4B* |  |  |  |  |  | ↓/↑* |  |  |  |  | [14] |
| *NtZIP4B* |  |  | ↑ |  |  |  | =/↑*** | ↓ |  |  | [13] |
| *NtZIP5A* |  |  | ↑ |  |  |  | = | - |  |  |  |
| *NtZIP5A***** | + | + | ↑ | ↑ | = | = |  |  |  |  | [2] |
| *NtZIP5B* |  |  | ↑ |  |  |  | =/↑*** | - | Zn, Cd | PM | [13] |
| *NtZIP5-like* | -/+* | -/+* | - | = | - | ↓ |  |  |  |  | [2] |
| *NtZIP5-like* |  |  |  |  |  | = |  |  |  |  | [14] |
| *NtZIP5-like* |  |  | ↑ |  |  |  | = | = |  |  | [13] |
| *NtZIP8* | + | + | ↑ | = | = | = |  |  |  |  | [2] |
| *NtZIP8* |  |  | ↑ |  |  |  | = | = |  |  | [13] |
| *NtZIP11* | + | + | = | = | = | ↑ |  |  | Zn | PM | [9], [14] |
| *NtZIP11* |  |  | = |  |  |  | =/↑*** | ↓ |  |  | [13] |
| *NtNRAMP1* | + | + |  |  | ↑/↓* | ↑/↓* | ↓/↑* | ↑/=* |  |  | [20] |
| *NtNRAMP1* |  |  |  |  |  |  |  |  | Fe, Cd | PM | [16] |
| *NtNRAMP2-like* |  |  |  |  |  | ↑ |  |  |  |  | [14] |
| *NtNRAMP3* |  |  |  |  |  |  | ↑ | ↑ |  |  | [22] |
| *NtNRAMP3* | + | + | = | = | = | = | = | = | Fe, Mn, Co, Cd, Cu, Ni, Zn | PM | [10] |
| *NtNRAMP3-like* |  |  |  |  |  | ↑ |  |  |  |  | [14] |
| *NtNRAMP5l* |  |  |  |  |  |  |  |  | Cd, Mn | PM | [19] |
| *NtNRAMP5s* |  |  |  |  |  |  |  |  |  | PM |  |
| *NtNRAMP6* |  |  |  |  |  |  | ↑ | ↑ |  |  | [22] |
| *NtNRAMP6-like* |  |  |  |  |  | = |  |  |  |  | [14] |
| *NgMTP1* |  |  |  |  |  |  |  |  | Zn, Co | V | [17] |
| *NtMTP1a* | + | + |  |  |  |  | ↑ | ↓ |  |  | [4] |
| *NtMTP1a* |  |  |  |  |  |  |  |  | Zn, Co | V | [3], [17] |
| *NtMTP1b* |  |  |  |  |  |  |  |  | Zn, Co | V | [17] |
| *NtMTP1* | + | + |  |  | = | ↓/↑* | = | ↑ |  |  | [20] |
| *NtMTP1* | + |  |  |  | ↑ |  |  |  |  |  | [3] |
| *NtMTP1.1* | + | + |  |  | = | = | = | ↑ |  | V | [12] |
| *NtMTP1.2* | + | + |  |  | ↑ | ↑ | ↑ | ↑ |  | V |  |
| *NtMTP2* | + | + |  |  |  | ↑ |  |  | Co, Ni | V | [14], [15] |
| *NtMTP4-like* |  |  |  |  |  | = |  |  |  |  | [14] |
| *NtMTP4.1* | + | + |  |  | ↓ | ↓ | ↓ | = |  | V | [12] |
| *NtMTP4.2* | + | + |  |  | ↓ | ↓ | ↓ | ↑ |  | V |  |
| *NtMTP5.1* | + | + |  |  | ↑ | = | ↑ | ↑ |  | V |  |
| *NtMTP5.2* | + | + |  |  | ↑ | ↑ | ↑ | ↑ |  | V |  |
| *NtMTP6.1* | + | + |  |  | = | ↑ | ↑ | ↑ |  | V/NUC |  |
| *NtMTP6.2* | + | + |  |  | = | ↓ | ↑ | = |  | V/NUC |  |
| *NtMTP7.1* | + | + |  |  | = | ↑ | ↑ | ↑ |  | V |  |
| *NtMTP7.2* | + | + |  |  | ↑ | ↑ | ↑ | ↑ |  | V |  |
| *NtMTP7.3* | + | + |  |  | ↓ | = | = | = |  | V |  |
| *NtMTP8.1* | + | + |  |  | ↑ | = | ↑ | = |  | V |  |
| *NtMTP8.2* | + | + |  |  | ↑ | ↑ | ↑ | ↑ |  | V |  |
| *NtMTP8.3* | + | - |  |  | ↑ | ↑ | ↑ | ↑ |  | V |  |
| *NtMTP8.4* | + | + |  |  | ↑ | ↑ | ↑ | ↑ |  | V |  |
| *NtMTP8.5* | + | + |  |  | ↑ | ↑ | = | ↑ |  | PM/V |  |
| *NtMTP9.1* | - | - |  |  | ↑ | = | ↑ | ↑ |  | V |  |
| *NtMTP9.2* | - | + |  |  | ↑ | = | ↓ | ↑ |  | V |  |
| *NtMTP10.1* | + | + |  |  | = | = | = | ↑ |  | PM/V |  |
| *NtMTP10.2* | + | + |  |  | ↓ | ↓ | ↓ | = |  | V |  |
| *NtMTP10.3* | + | + |  |  | ↑ | ↑ | ↑ | ↑ |  | V |  |
| *NtMTP10.4* | + | + |  |  | ↑ | = | ↑ | ↑ |  | PM/V |  |
| *NtMTP11.1* | + | + |  |  | ↑ | ↑ | ↑ | ↑ |  | V |  |
| *NtMTP11.2* | + | + |  |  | ↑ | ↑ | ↑ | ↑ |  | V |  |
| *NtMTP12.1* | + | + |  |  | = | = | ↑ | = |  | V |  |
| *NtMTP12.2* | + | + |  |  | ↑ | ↓ | ↑ | = |  | V |  |
| *NtMTPC2-like* |  |  |  |  |  | ↑ |  |  |  |  | [14] |
| *NtHMAα* | + | + |  |  | ↑ |  | ↑ |  | Zn, Cd |  | [6] |
| *NtHMAβ* | + | + |  |  | ↑ |  | ↑ |  | Zn, Cd |  |  |
| *NtHMA2α* |  |  |  |  |  |  | = | ↑ |  |  | [22] |
| *NtHMA2β* |  |  |  |  |  |  | ↑ | ↑ |  |  |  |
| *NtHMA3* | - | - |  |  |  |  | ↑ | - |  |  | [4] |
| *NtHMA4* |  |  |  |  |  |  | ↑ | = |  |  | [22] |
| *NtYSL1* | - |  |  |  |  |  | ↑ |  |  |  | [7] |
| *NtYSL1* |  |  |  |  |  |  | ↑ | ↑ |  |  | [22] |
| *NtYSL6* |  |  |  |  |  |  | ↑ | ↑ |  |  |  |
| *NtYSL7* |  |  |  |  |  |  | ↑ | ↑ |  |  |  |
| *NtMRP2-like* |  |  |  |  |  | ↑ |  |  |  |  | [14] |
| *NtMRP3-like* |  |  |  |  |  | ↑ |  |  |  |  |  |
| *NtMRP5-like* |  |  |  |  |  | ↑ |  |  |  |  |  |
| *NtMRP10-like* |  |  |  |  |  | ↑ |  |  |  |  |  |
| *NtMRP14-like* |  |  |  |  |  | ↑ |  |  |  |  |  |
| *NtMRP15-like* |  |  |  |  |  | = |  |  |  |  |  |
| *NtCBP4* |  |  |  |  |  |  |  |  | Ni, Pb | PM | [1], [18] |
| *NtFRO1* |  | + |  |  |  |  |  |  |  |  | [5] |
| *NtFRO1*** | +** | +** |  |  |  |  | =/↑** | =/↑** |  |  | [21] |
| *NtNAS3* | + | - |  |  |  |  | ↑ | - |  |  | [4] |
| *NtPCS1* |  |  |  |  |  |  |  |  | Cd, As | TP | [8], [11] |

**Supplementary Table S1.** Regulation of discussed tobacco genes by the deficiency or excess of selected metals (continued).

FRO - ferric chelate reductase gene; HMA – genes encoding Heavy Metal Associated Proteins; MRP - genes encoding Multidrag Resistance Proteins; MTP - genes encoding Metal Tolerance Proteins; NAS – gene encoding Nicotianamine Synthase; NRAMP - genes encoding Natural Resistance-Associated Macrophage Proteins; YSL - genes encoding Yellow Stripe-Like Proteins; ZIP - genes encoding Zrt-/Irt-like Proteins; SB – substrates; SL – subcellular localization; NUC – nucleus; PM – plasma membrane; V – vacuole; R – roots; S/L – shoot/leaves; + transcript was present; - transcript was not detected; ↓ downregulation; ↑ upregulation; = control level; * expression depends on the plant age; ** detected in whole seedlings; *** expression depends on the root fragment, Cd concentration and time after treatment; **** the transcript was renamed from NtZIP1 to NtZIP5A according to Palusińska et al. 2020; empty boxes – expression was not determined.

| **GENE** | **METAL STATUS** | | | | | | | | | | | | **SB** | **SL** | **REF.** |  |
| --- | --- | --- | --- | --- | --- | --- | --- | --- | --- | --- | --- | --- | --- | --- | --- | --- |
|  | **Control** | | **Fe** | | | | **Co** | | | | **Ni** | |  |  |  |  |
|  |  |  | **deficiency** | | **excess** | | **deficiency** | | **excess** | | **excess** | |  |  |  |  |
|  | **R** | **S/L** | **R** | **S/L** | **R** | **S/L** | **R** | **S/L** | **R** | **S/L** | **R** | **S/L** |  |  |  | |
| *NtIRT1* | + | - |  |  |  |  |  |  |  |  |  |  |  |  | [2] | |
| *NtIRT1* |  |  |  |  |  |  |  |  |  |  |  |  |  |  | [13] | |
| *NtIRT1* | - | - |  |  |  |  |  |  |  |  |  |  |  |  | [4] | |
| *NtIRT1* | + | + | ↑ | ↑ |  |  |  |  |  |  |  |  |  |  | [5] | |
| *NtIRT1* | - |  | ↑ |  |  |  |  |  |  |  |  |  |  |  | [7] | |
| *NtIRT1*** | +** | +** | ↑** | ↑** |  |  |  |  |  |  |  |  |  |  | [21] | |
| *NtIRT1-like* | + | - |  |  |  |  |  |  |  |  |  |  |  |  | [2] | |
| *NtIRT1-like* |  |  |  |  |  |  |  |  |  |  |  |  |  |  | [13] | |
| *NtZIP1* | + | -/+* |  |  |  |  |  |  |  |  |  |  |  |  | [20] | |
| *NtZIP1* |  |  |  |  |  |  |  |  |  |  |  |  |  |  | [22] | |
| *NtZIP1-like* | + | + |  |  |  |  |  |  |  |  |  |  | Zn | PM | [14] | |
| *NtZIP1-like* |  |  |  |  |  |  |  |  |  |  |  |  |  |  | [13] | |
| *NtZIP2* | + | - |  |  |  |  |  |  |  |  |  |  |  |  | [2] | |
| *NtZIP2* |  |  |  |  |  |  |  |  |  |  |  |  |  |  | [13] | |
| *NtZIP3* |  |  |  |  |  |  |  |  |  |  |  |  |  |  | [22] | |
| *NtZIP4* |  |  |  |  |  |  |  |  |  |  |  |  |  |  |  |  |
| *NtZIP4A* | + | + |  |  |  |  |  |  |  |  |  |  |  |  | [2] | |
| *NtZIP4A* |  |  |  |  |  |  |  |  |  |  |  |  |  |  | [13] | |
| *NtZIP4B* | + | + |  |  |  |  |  |  |  |  |  |  | Zn, Cd | PM | [2] | |
| *NtZIP4B* |  |  |  |  |  |  |  |  |  |  |  |  |  |  | [14] | |
| *NtZIP4B* |  |  |  |  |  |  |  |  |  |  |  |  |  |  | [13] | |
| *NtZIP5A* |  |  |  |  |  |  |  |  |  |  |  |  |  |  |  |  |
| *NtZIP5A***** | + | + |  |  |  |  |  |  |  |  |  |  |  |  | [2] | |
| *NtZIP5B* |  |  |  |  |  |  |  |  |  |  |  |  | Zn, Cd | PM | [13] | |
| *NtZIP5-like* | -/+* | -/+* |  |  |  |  |  |  |  |  |  |  |  |  | [2] | |
| *NtZIP5-like* |  |  |  |  |  |  |  |  |  |  |  |  |  |  | [14] | |
| *NtZIP5-like* |  |  |  |  |  |  |  |  |  |  |  |  |  |  | [13] | |
| *NtZIP8* | + | + |  |  |  |  |  |  |  |  |  |  |  |  | [2] | |
| *NtZIP8* |  |  |  |  |  |  |  |  |  |  |  |  |  |  | [13] | |
| *NtZIP11* | + | + |  |  |  |  |  |  |  |  |  |  | Zn | PM | [9], [14] | |
| *NtZIP11* |  |  |  |  |  |  |  |  |  |  |  |  |  |  | [13] | |
| *NtNRAMP1* | + | + |  |  |  |  |  |  |  |  |  |  |  |  | [20] | |
| *NtNRAMP1* |  |  |  |  |  |  |  |  |  |  |  |  | Fe, Cd | PM | [16] | |
| *NtNRAMP2-like* |  |  |  |  |  |  |  |  |  |  |  |  |  |  | [14] | |
| *NtNRAMP3* |  |  |  |  |  |  |  |  |  |  |  |  |  |  | [22] | |
| *NtNRAMP3* | + | + | ↓ | ↓ | ↓ | ↓ | ↓ | ↓ | ↓ | ↓ | ↓ | ↓ | Fe, Mn, Co, Cd, Cu, Ni, Zn | PM | [10] | |
| *NtNRAMP3-like* |  |  |  |  |  |  |  |  |  |  |  |  |  |  | [14] | |
| *NtNRAMP5l* |  |  |  |  |  |  |  |  |  |  |  |  | Cd, Mn | PM | [19] | |
| *NtNRAMP5s* |  |  |  |  |  |  |  |  |  |  |  |  |  | PM |  |  |
| *NtNRAMP6* |  |  |  |  |  |  |  |  |  |  |  |  |  |  | [22] | |
| *NtNRAMP6-like* |  |  |  |  |  |  |  |  |  |  |  |  |  |  | [14] | |
| *NgMTP1* |  |  |  |  |  |  |  |  |  |  |  |  | Zn, Co | V | [17] | |
| *NtMTP1a* | + | + |  |  |  |  |  |  |  |  |  |  |  |  | [4] | |
| *NtMTP1a* |  |  |  |  |  |  |  |  |  |  |  |  | Zn, Co | V | [3], [17] | |
| *NtMTP1b* |  |  |  |  |  |  |  |  |  |  |  |  | Zn, Co | V | [17] | |
| *NtMTP1* | + | + |  |  |  |  |  |  |  |  |  |  |  |  | [20] | |
| *NtMTP1* | + |  |  |  |  |  |  |  |  |  |  |  |  |  | [3] | |
| *NtMTP1.1* | + | + |  |  | = | = |  |  | = | = |  |  |  | V | [12] | |
| *NtMTP1.2* | + | + |  |  | ↓ | = |  |  | ↑ | ↑ |  |  |  | V |  |  |
| *NtMTP2* | + | + |  |  |  |  |  |  | = | ↑ | = | = | Co, Ni | V | [14], [15] | |
| *NtMTP4-like* |  |  |  |  |  |  |  |  |  |  |  |  |  |  | [14] | |
| *NtMTP4.1* | + | + |  |  | = | ↓ |  |  | = | ↓ |  |  |  | V | [12] | |
| *NtMTP4.2* | + | + |  |  | = | ↓ |  |  | = | ↓ |  |  |  | V |  |  |
| *NtMTP5.1* | + | + |  |  | = | = |  |  | ↑ | = |  |  |  | V |  |  |
| *NtMTP5.2* | + | + |  |  | = | = |  |  | ↑ | = |  |  |  | V |  |  |
| *NtMTP6.1* | + | + |  |  | = | = |  |  | ↑ | = |  |  |  | V/NUC |  |  |
| *NtMTP6.2* | + | + |  |  | = | ↓ |  |  | ↑ | = |  |  |  | V/NUC |  |  |
| *NtMTP7.1* | + | + |  |  | ↓ | = |  |  | ↑ | ↑ |  |  |  | V |  |  |
| *NtMTP7.2* | + | + |  |  | = | = |  |  | ↑ | ↑ |  |  |  | V |  |  |
| *NtMTP7.3* | + | + |  |  | = | ↓ |  |  | ↑ | = |  |  |  | V |  |  |
| *NtMTP8.1* | + | + |  |  | ↑ | = |  |  | ↑ | = |  |  |  | V |  |  |
| *NtMTP8.2* | + | + |  |  | ↑ | ↓ |  |  | ↑ | ↑ |  |  |  | V |  |  |
| *NtMTP8.3* | + | - |  |  | ↓ | = |  |  | ↑ | ↓ |  |  |  | V |  |  |
| *NtMTP8.4* | + | + |  |  | = | = |  |  | ↑ | ↑ |  |  |  | V |  |  |
| *NtMTP8.5* | + | + |  |  | ↑ | ↓ |  |  | ↑ | ↑ |  |  |  | PM/V |  |  |
| *NtMTP9.1* | - | - |  |  | = | = |  |  | ↑ | ↑ |  |  |  | V |  |  |
| *NtMTP9.2* | - | + |  |  | ↓ | = |  |  | ↓ | = |  |  |  | V |  |  |
| *NtMTP10.1* | + | + |  |  | ↓ | ↑ |  |  | ↑ | ↓ |  |  |  | PM/V |  |  |
| *NtMTP10.2* | + | + |  |  | ↓ | ↓ |  |  | ↑ | ↑ |  |  |  | V |  |  |
| *NtMTP10.3* | + | + |  |  | ↑ | ↑ |  |  | ↑ | = |  |  |  | V |  |  |
| *NtMTP10.4* | + | + |  |  | = | ↑ |  |  | ↑ | = |  |  |  | PM/V |  |  |
| *NtMTP11.1* | + | + |  |  | ↑ | ↓ |  |  | ↑ | ↑ |  |  |  | V |  |  |
| *NtMTP11.2* | + | + |  |  | ↑ | = |  |  | ↑ | ↑ |  |  |  | V |  |  |
| *NtMTP12.1* | + | + |  |  | ↓ | = |  |  | ↑ | = |  |  |  | V |  |  |
| *NtMTP12.2* | + | + |  |  | ↓ | ↓ |  |  | ↑ | = |  |  |  | V |  |  |
| *NtMTPC2-like* |  |  |  |  |  |  |  |  |  |  |  |  |  |  | [14] | |
| *NtHMAα* | + | + |  |  |  |  |  |  |  |  |  |  | Zn, Cd |  | [6] | |
| *NtHMAβ* | + | + |  |  |  |  |  |  |  |  |  |  | Zn, Cd |  |  |  |
| *NtHMA2α* |  |  |  |  |  |  |  |  |  |  |  |  |  |  | [22] | |
| *NtHMA2β* |  |  |  |  |  |  |  |  |  |  |  |  |  |  |  |  |
| *NtHMA3* | - | - |  |  |  |  |  |  |  |  |  |  |  |  | [4] | |
| *NtHMA4* |  |  |  |  |  |  |  |  |  |  |  |  |  |  | [22] | |
| *NtYSL1* | - |  | ↑ |  |  |  |  |  |  |  |  |  |  |  | [7] | |
| *NtYSL1* |  |  |  |  |  |  |  |  |  |  |  |  |  |  | [22] | |
| *NtYSL6* |  |  |  |  |  |  |  |  |  |  |  |  |  |  |  |  |
| *NtYSL7* |  |  |  |  |  |  |  |  |  |  |  |  |  |  |  |  |
| *NtMRP2-like* |  |  |  |  |  |  |  |  |  |  |  |  |  |  | [14] | |
| *NtMRP3-like* |  |  |  |  |  |  |  |  |  |  |  |  |  |  |  |  |
| *NtMRP5-like* |  |  |  |  |  |  |  |  |  |  |  |  |  |  |  |  |
| *NtMRP10-like* |  |  |  |  |  |  |  |  |  |  |  |  |  |  |  |  |
| *NtMRP14-like* |  |  |  |  |  |  |  |  |  |  |  |  |  |  |  |  |
| *NtMRP15-like* |  |  |  |  |  |  |  |  |  |  |  |  |  |  |  |  |
| *NtCBP4* |  |  |  |  |  |  |  |  |  |  |  |  | Ni, Pb | PM | [1], [18] | |
| *NtFRO1* |  | + | ↑ |  |  |  |  |  |  |  |  |  |  |  | [5] | |
| *NtFRO1*** | +** | +** | ↑** | ↑** |  |  |  |  |  |  |  |  |  |  | [21] | |
| *NtNAS3* | + | - |  |  |  |  |  |  |  |  |  |  |  |  | [4] | |
| *NtPCS1* |  |  |  |  |  |  |  |  |  |  |  |  | Cd, As | TP | [8], [11] | |

**Supplementary Table S1.** Regulation of discussed tobacco genes by the deficiency or excess of selected metals (continued).

FRO - ferric chelate reductase gene; HMA – genes encoding Heavy Metal Associated Proteins; MRP - genes encoding Multidrag Resistance Proteins; MTP - genes encoding Metal Tolerance Proteins; NAS – gene encoding Nicotianamine Synthase; NRAMP - genes encoding Natural Resistance-Associated Macrophage Proteins; YSL - genes encoding Yellow Stripe-Like Proteins; ZIP - genes encoding Zrt-/Irt-like Proteins; SB – substrates; SL – subcellular localization; NUC – nucleus; PM – plasma membrane; V – vacuole; R – roots; S/L – shoot/leaves; + transcript was present; - transcript was not detected; ↓ downregulation; ↑ upregulation; = control level; * expression depends on the plant age; ** detected in whole seedlings; *** expression depends on the root fragment, Cd concentration and time after treatment; **** the transcript was renamed from NtZIP1 to NtZIP5A according to Palusińska et al. 2020; empty boxes – expression was not determined.

| **GENE** | **METAL STATUS** | | | | | | | | **SB** | **SL** | **REF.** |  |
| --- | --- | --- | --- | --- | --- | --- | --- | --- | --- | --- | --- | --- |
|  | **Control** | | **Mn** | | | | **Mg** | |  |  |  |  |
|  |  |  | **deficiency** | | **excess** | | **excess** | |  |  |  |  |
|  | **R** | **S/L** | **R** | **S/L** | **R** | **S/L** | **R** | **S/L** |  |  |  | |
| *NtIRT1* | + | - |  |  |  |  |  |  |  |  | v | |
| *NtIRT1* |  |  |  |  |  |  |  |  |  |  | [12] | |
| *NtIRT1* | - | - |  |  |  |  |  |  |  |  | [4] | |
| *NtIRT1* | + | + |  |  |  |  |  |  |  |  | [5] | |
| *NtIRT1* | - |  |  |  |  |  |  |  |  |  | [7] | |
| *NtIRT1*** | +** | +** |  |  |  |  |  |  |  |  | [20] | |
| *NtIRT1-like* | + | - |  |  |  |  |  |  |  |  | [2] | |
| *NtIRT1-like* |  |  |  |  |  |  |  |  |  |  | [12] | |
| *NtZIP1* | + | -/+* |  |  |  |  |  |  |  |  | [19] | |
| *NtZIP1* |  |  |  |  |  |  |  |  |  |  | [21] | |
| *NtZIP1-like* | + | + |  |  |  |  |  |  | Zn | PM | [13] | |
| *NtZIP1-like* |  |  |  |  |  |  |  |  |  |  | [12] | |
| *NtZIP2* | + | - |  |  |  |  |  |  |  |  | [2] | |
| *NtZIP2* |  |  |  |  |  |  |  |  |  |  | [12] | |
| *NtZIP3* |  |  |  |  |  |  |  |  |  |  | [21] | |
| *NtZIP4* |  |  |  |  |  |  |  |  |  |  |  |  |
| *NtZIP4A* | + | + |  |  |  |  |  |  |  |  | [2] | |
| *NtZIP4A* |  |  |  |  |  |  |  |  |  |  | [12] | |
| *NtZIP4B* | + | + |  |  |  |  |  |  | Zn, Cd | PM | [2] | |
| *NtZIP4B* |  |  |  |  |  |  |  |  |  |  | [13] | |
| *NtZIP4B* |  |  |  |  |  |  |  |  |  |  | [12] | |
| *NtZIP5A* |  |  |  |  |  |  |  |  |  |  |  |  |
| *NtZIP5A***** | + | + |  |  |  |  |  |  |  |  | [2] | |
| *NtZIP5B* |  |  |  |  |  |  |  |  | Zn, Cd | PM | [12] | |
| *NtZIP5-like* | -/+* | -/+* |  |  |  |  |  |  |  |  | [2] | |
| *NtZIP5-like* |  |  |  |  |  |  |  |  |  |  | [13] | |
| *NtZIP5-like* |  |  |  |  |  |  |  |  |  |  | [12] | |
| *NtZIP8* | + | + |  |  |  |  |  |  |  |  | [2] | |
| *NtZIP8* |  |  |  |  |  |  |  |  |  |  | [12] | |
| *NtZIP11* | + | + |  |  |  |  |  |  | Zn | PM | [9], [13] | |
| *NtZIP11* |  |  |  |  |  |  |  |  |  |  | [12] | |
| *NtNRAMP1* | + | + |  |  |  |  |  |  |  |  | [19] | |
| *NtNRAMP1* |  |  |  |  |  |  |  |  | Fe, Cd | PM | [15] | |
| *NtNRAMP2-like* |  |  |  |  |  |  |  |  |  |  | [13] | |
| *NtNRAMP3* |  |  |  |  |  |  |  |  |  |  | [21] | |
| *NtNRAMP3* | + | + | ↓ | ↓ | ↓ | ↓ |  |  | Fe, Mn, Co, Cd, Cu, Ni, Zn | PM | [10] | |
| *NtNRAMP3-like* |  |  |  |  |  |  |  |  |  |  | [13] | |
| *NtNRAMP5l* |  |  |  |  |  |  |  |  | Cd, Mn | PM | [18] | |
| *NtNRAMP5s* |  |  |  |  |  |  |  |  |  | PM |  |  |
| *NtNRAMP6* |  |  |  |  |  |  |  |  |  |  | [21] | |
| *NtNRAMP6-like* |  |  |  |  |  |  |  |  |  |  | [13] | |
| *NgMTP1* |  |  |  |  |  |  |  |  | Zn, Co | V | [16] | |
| *NtMTP1a* | + | + |  |  |  |  |  |  |  |  | [4] | |
| *NtMTP1a* |  |  |  |  |  |  |  |  | Zn, Co | V | [3], [16] | |
| *NtMTP1b* |  |  |  |  |  |  |  |  | Zn, Co | V | [16] | |
| *NtMTP1* | + | + |  |  |  |  |  |  |  |  | [19] | |
| *NtMTP1* | + |  |  |  |  |  |  |  |  |  | [3] | |
| *NtMTP1.1* | + | + |  |  | = | = | = | = |  | V | [11] | |
| *NtMTP1.2* | + | + |  |  | ↑ | = | = | = |  | V |  |  |
| *NtMTP2* | + | + |  |  |  |  |  |  | Co, Ni | V | [13], [14] | |
| *NtMTP4-like* |  |  |  |  |  |  |  |  |  |  | [13] | |
| *NtMTP4.1* | + | + |  |  | ↓ | ↓ | = | = |  | V | [11] | |
| *NtMTP4.2* | + | + |  |  | ↓ | ↓ | = | = |  | V |  |  |
| *NtMTP5.1* | + | + |  |  | ↓ | = | = | ↑ |  | V |  |  |
| *NtMTP5.2* | + | + |  |  | ↓ | = | = | ↑ |  | V |  |  |
| *NtMTP6.1* | + | + |  |  | = | = | = | = |  | V/NUC |  |  |
| *NtMTP6.2* | + | + |  |  | = | ↓ | ↑ | ↓ |  | V/NUC |  |  |
| *NtMTP7.1* | + | + |  |  | = | = | = | = |  | V |  |  |
| *NtMTP7.2* | + | + |  |  | = | ↑ | = | ↑ |  | V |  |  |
| *NtMTP7.3* | + | + |  |  | = | = | = | = |  | V |  |  |
| *NtMTP8.1* | + | + |  |  | = | ↓ | = | = |  | V |  |  |
| *NtMTP8.2* | + | + |  |  | ↑ | = | ↑ | ↑ |  | V |  |  |
| *NtMTP8.3* | + | - |  |  | ↑ | ↑ | = | = |  | V |  |  |
| *NtMTP8.4* | + | + |  |  | ↑ | ↑ | = | ↑ |  | V |  |  |
| *NtMTP8.5* | + | + |  |  | ↑ | = | = | ↑ |  | PM/V |  |  |
| *NtMTP9.1* | - | - |  |  | ↑ | ↓ | = | = |  | V |  |  |
| *NtMTP9.2* | - | + |  |  | ↓ | = | ↓ | = |  | V |  |  |
| *NtMTP10.1* | + | + |  |  | = | = | ↓ | = |  | PM/V |  |  |
| *NtMTP10.2* | + | + |  |  | ↑ | ↓ | ↑ | ↓ |  | V |  |  |
| *NtMTP10.3* | + | + |  |  | ↑ | = | = | = |  | V |  |  |
| *NtMTP10.4* | + | + |  |  | ↑ | ↑ | = | ↑ |  | PM/V |  |  |
| *NtMTP11.1* | + | + |  |  | ↓ | ↑ | ↓ | ↑ |  | V |  |  |
| *NtMTP11.2* | + | + |  |  | ↓ | ↑ | = | ↑ |  | V |  |  |
| *NtMTP12.1* | + | + |  |  | = | ↓ | = | = |  | V |  |  |
| *NtMTP12.2* | + | + |  |  | = | ↓ | = | = |  | V |  |  |
| *NtMTPC2-like* |  |  |  |  |  |  |  |  |  |  | [13] | |
| *NtHMAα* | + | + |  |  |  |  |  |  | Zn, Cd |  | [6] | |
| *NtHMAβ* | + | + |  |  |  |  |  |  | Zn, Cd |  |  |  |
| *NtHMA2α* |  |  |  |  |  |  |  |  |  |  | [21] | |
| *NtHMA2β* |  |  |  |  |  |  |  |  |  |  |  |  |
| *NtHMA3* | - | - |  |  |  |  |  |  |  |  | [4] | |
| *NtHMA4* |  |  |  |  |  |  |  |  |  |  | [21] | |
| *NtYSL1* | - |  |  |  |  |  |  |  |  |  | [7] | |
| *NtYSL1* |  |  |  |  |  |  |  |  |  |  | [21] | |
| *NtYSL6* |  |  |  |  |  |  |  |  |  |  |  |  |
| *NtYSL7* |  |  |  |  |  |  |  |  |  |  |  |  |
| *NtMRP2-like* |  |  |  |  |  |  |  |  |  |  | [13] | |
| *NtMRP3-like* |  |  |  |  |  |  |  |  |  |  |  |  |
| *NtMRP5-like* |  |  |  |  |  |  |  |  |  |  |  |  |
| *NtMRP10-like* |  |  |  |  |  |  |  |  |  |  |  |  |
| *NtMRP14-like* |  |  |  |  |  |  |  |  |  |  |  |  |
| *NtMRP15-like* |  |  |  |  |  |  |  |  |  |  |  |  |
| *NtCBP4* |  |  |  |  |  |  |  |  | Ni, Pb | PM | [1], [18] | |
| *NtFRO1* |  | + |  |  |  |  |  |  |  |  | [5] | |
| *NtFRO1*** | +** | +** |  |  |  |  |  |  |  |  | [21] | |
| *NtNAS3* | + | - |  |  |  |  |  |  |  |  | [4] | |
| *NtPCS1* |  |  |  |  |  |  |  |  | Cd, As | TP | [8], [11] | |

**References:**

1. Arazi T, Sunkar R, Kaplan B, Fromm H (1999) A tobacco plasma membrane calmodulin‐binding transporter confers Ni2+ tolerance and Pb2+ hypersensitivity in transgenic plants. Plant J 20(2):171-182. https://doi.org/10.1046/j.1365-313x.1999.00588.x
2. Barabasz A, Palusińska M, Papierniak A, Kendziorek M, Kozak K, Williams LE, Antosiewicz, DM (2019) Functional analysis of NtZIP4B and Zn status-dependent expression pattern of tobacco ZIP genes. Front Plant Sci 9:1984. https://doi.org/10.3389/fpls.2018.01984
3. Bazihizina N, Taiti C, Marti L, Rodrigo-Moreno A, Spinelli F, Giordano C et al (2014) Zn2+-induced changes at the root level account for the increased tolerance of acclimated tobacco plants. J Exp Bot 65(17):4931-4942. https://doi.org/10.1093/jxb/eru251
4. Bovet L, Rossi L, Lugon‐Moulin N (2006) Cadmium partitioning and gene expression studies in Nicotiana tabacum and Nicotiana rustica. Physiol Plant 128(3):466-475. https://doi.org/10.1111/j.1399-3054.2006.00756.x
5. Enomoto Y, Hodoshima H, Shimada H, Shoji K, Yoshihara T, Goto F (2007) Long-distance signals positively regulate the expression of iron uptake genes in tobacco roots. Planta 227(1):81-89. https://doi.org/10.1007/s00425-007-0596-x
6. Hermand V, Julio E, Dorlhac de Borne F, Punshon T, Ricachenevsky FK, Bellec A et al (2014) Inactivation of two newly identified tobacco heavy metal ATPases leads to reduced Zn and Cd accumulation in shoots and reduced pollen germination. Metallomics 6(8):1427-1440. https://doi.org/10.1039/c4mt00071d
7. Hodoshima H, Enomoto Y, Shoji K, Shimada H, Goto F, Yoshihara T (2007) Differential regulation of cadmium‐inducible expression of iron‐deficiency‐responsive genes in tobacco and barley. Physiol Plant 129(3):622-634. https://doi.org/10.1111/j.1399-3054.2006.00825.x
8. Kim YJ, Chang KS, Lee MR, Kim JH, Lee CE, Jeon YJ et al (2005) Expression of tobacco cDNA encoding phytochelatin synthase promotes tolerance to and accumulation of Cd and As in Saccharomyces cerevisiae. J Plant Biol 48(4):440-447. https://doi.org/10.1007/BF03030586
9. Kozak K, Papierniak A, Barabasz A, Kendziorek M, Palusińska M, Williams LE, Antosiewicz DM (2019) NtZIP11, a new Zn transporter specifically upregulated in tobacco leaves by toxic Zn level. Environ Exp Bot 157:69-78. https://doi.org/10.1016/j.envexpbot.2018.09.020
10. Kozak K, Papierniak-Wygladala A, Palusińska M, Barabasz A, Antosiewicz DM (2022) Regulation and Function of Metal Uptake Transporter NtNRAMP3 in Tobacco. Front Plant Sci 13:867967. https://doi.org/10.3389/fpls.2022.867967
11. Lee BD, Hwang S (2015) Tobacco phytochelatin synthase (NtPCS1) plays important roles in cadmium and arsenic tolerance and in early plant development in tobacco. Plant Biotechnol Rep 9(3):107-114. https://doi.org/10.1007/s11816-015-0348-5
12. Liu J, Gao Y, Tang Y, Wang D, Chen X, Yao Y, Guo Y. (2019) Genome-wide identification, comprehensive gene feature, evolution, and expression analysis of plant metal tolerance proteins in tobacco under heavy metal toxicity. Front Genet 10:345. https://doi.org/10.3389/fgene.2019.00345
13. Palusińska M, Barabasz A, Kozak K, Papierniak A, Maślińska K, Antosiewicz DM (2020) Zn/Cd status-dependent accumulation of Zn and Cd in root parts in tobacco is accompanied by specific expression of ZIP genes. BMC Plant Biol 20(1):1-19. https://doi.org/10.1186/s12870-020-2255-3
14. Papierniak A, Kozak K, Kendziorek M, Barabasz A, Palusińska M, Tiuryn, J et al (2018) Contribution of NtZIP1-Like to the regulation of Zn homeostasis. Front Plant Sci 9:185. https://doi.org/10.3389/fpls.2018.00185
15. Papierniak-Wygladala A, Kozak K, Barabasz A, Palusińska M, Całka M, Maślińska K, Antosiewicz DM (2020) Identification and characterization of a tobacco metal tolerance protein, NtMTP2. Metallomics 12(12):2049-2064. https://doi.org/10.1039/d0mt00210k
16. Sano T, Yoshihara T, Handa K, Sato MH, Nagata T, Hasezawa S (2012) Metal ion homeostasis mediated by Nramp transporters in plant cells-focused on increased resistance to iron and cadmium ion. In: Crosstalk and integration of membrane trafficking pathways, p. 213-228.
17. Shingu Y, Kudo T, Ohsato S, Kimura M, Ono Y, Yamaguchi I, Hamamoto H (2005) Characterization of genes encoding metal tolerance proteins isolated from Nicotiana glauca and Nicotiana tabacum. Biochem Biophys Res Commun 331:675-680. https://doi.org/10.1016/j.bbrc.2005.04.010
18. Sunkar R, Kaplan B, Bouché N, Arazi T, Dolev D, Talke IN et al (2000) Expression of a truncated tobacco NtCBP4 channel in transgenic plants and disruption of the homologous Arabidopsis CNGC1 gene confer Pb2+ tolerance. Plant J 24:533-542. https://doi.org/10.1111/j.1365-313X.2000.00901.x
19. Tang Z, Cai H, Li J, Lv Y, Zhang W, Zhao FJ (2017) Allelic variation of NtNramp5 associated with cultivar variation in cadmium accumulation in tobacco. Plant Cell Physiol 58(9):1583-1593. https://doi.org/10.1093/pcp/pcx087
20. Vera-Estrella R, Gómez-Méndez MF, Amezcua-Romero JC, Barkla BJ, Rosas-Santiago P, Pantoja O (2017) Cadmium and zinc activate adaptive mechanisms in Nicotiana tabacum similar to those observed in metal tolerant plants. Planta 246(3):433-451. https://doi.org/10.1007/s00425-017-2700-1
21. Yoshihara T, Hodoshima H, Miyano Y, Shoji K, Shimada H, Goto F (2006) Cadmium inducible Fe deficiency responses observed from macro and molecular views in tobacco plants. Plant Cell Rep 25(4):365-373. https://doi.org/10.1007/s00299-005-0092-3
22. Zhang H, Lu X, Wang Z, Yan X, Cui H (2021) Excretion from long glandular trichomes contributes to alleviation of cadmium toxicity in Nicotiana tabacum. Environ Pollut 285:117184. https://doi.org/10.1016/j.envpol.2021.117184
