# Supplementary material for: Tobacco as an efficient metal accumulator
Source: Biometals. 2022 Sep 12;36(2):351–70. doi: 10.1007/s10534-022-00431-3 (PMC10082116; doi:10.1007/s10534-022-00431-3)
Supplement: Supplementary file 3 — Supplementary file3 (DOCX 54 kb)—Genes used for genetic modifications of tobacco [file 10534_2022_431_MOESM3_ESM.docx]

Article title: **Tobacco as an efficient metal accumulator**

Journal name: Biometals

Author names: Katarzyna Kozak and Danuta Maria Antosiewicz

Affiliation: University of Warsaw, Faculty of Biology, Institute of Experimental Plant Biology and Biotechnology, 1 Miecznikowa Str. 02-096 Warszawa, Poland

E-mail Address of the Corresponding Author: dma@biol.uw.edu.pl

**Supplementary Table S2**

**Supplementary Table S2.** A list of genes used for tobacco transformation to change metal transport properties.

Abbreviations: 1-CB - 1-Chlorobutane; 35Sp - a Long Version of the Cauliflower Mosaic Virus 35S Promoter; CaMV 35S - Cauliflower Mosaic Virus Promoter; *AhHMA4prom* – Native *AhHMA4* promoter; CT – Chlortoluron; DCA - 1,2-Dichloroethane; GDN - Glycerol Dinitrate; GMN - Glycerol Mononitrate; GNT - Glycerol Trinitrate; GSH – Glutathione; HCHO - Formaldehyde; IAA - Indole-3-acetic acid; ISP – Isoproturon; LN – Linuron; MMV - Mirabilis Mosaic Virus Promoter; NPT - Non-protein Thiols; NT – Non-transformed Plants; *NtREL1* - Root-Specific Promoter from a Tobacco Root Extension-like Protein-coding Gene; PC – Phytochelatins; PCP - Pentachlorophenol; *SRS1p* - Light-Induced Soybean Rubisco Promoter; TCE - Trichloroethylene TNT - 2,4,6-Trinitrotoluene; TP – Transgenic plants.

| **Gene(s)** | **Gene source** | | **Target plant** | **Promoter** | **Metal** | **Performance/Results** | **Reference** |
| --- | --- | --- | --- | --- | --- | --- | --- |
| 1. **Heterologous expression of metal transporters** | | | | | | | |
| *ArsC* | *Escherichia coli* | | *N. tabacum* | *SRS1p*, 35Sp | Cd | TP: higher tolerance to Cd; 30-50% higher Cd concentration than in NT plants  On medium with 50, 75, or 100 µM Cd:   1. TP - bigger with broader leaves and longer roots; 2. NT - stunted, turned yellow, flowered early and often died | Dhankher et al. 2003 |
| *AtCAX2* | *Arabidopsis thaliana* | | *N. tabacum* | CaMV 35S | Cd, Mn, Ca | TP: accumulated more Ca^2+^, Cd^2+^ and Mn^2+^; exhibited higher tolerance to elevated Mn^2+^ levels; 15 -20% more metal ions in shoot  Isolated tonoplast vesicles from roots of TP: increased Cd^2+^ and Mn^2+^ transport | Hirschi et al. 2000 |
| *AtCAX2* | *Arabidopsis thaliana* | | *N. tabacum* | CaMV 35S | Cd, Zn, Mn | TP grown on medium supplemented with 3 μM Cd^2+^, 500 μM Mn^2+^ or 150 μM Zn^2+^: higher dry weights and amount of metals accumulated in the roots and shoot | Korenkov et al. 2007 |
| *AtCAX4* | *Arabidopsis thaliana* | | *N. tabacum* | CaMV 35S | Cd, Zn, Mn |  |  |
| *AtECA3* | *Arabidopsis thaliana* | | *N. tabacum* | CaMV 35S | Cd, Mn, Ca | TP: higher Cd content in the roots (1 out of 6 lines); no changes in Cd content in the shoot; better growth on medium with 2 μM Mn (moderate concentration); TP grown on medium with 100 μM Mn: enhanced tolerance to Mn; enhanced tolerance to Ca deficiency; no changes in overall Ca content | Barabasz et al. 2011 |
| *AtMHX1* | *Arabidopsis thaliana* | | *N. tabacum* | CaMV 35S | Zn, Mg | TP: more sensitive to media containing elevated Mg and Zn; no changes in Mg and Zn content in shoot | Shaul et al. 1999 |
| *AtMHX* | *Arabidopsis thaliana* | | *N. tabacum* | CaMV 35S | Cd, Zn, Mg | TP grown on medium with increased concentrations of Mg^2+^, Zn^2+^ and Cd^2+^: developed necrotic lesions and apical burnings; smaller than NT plants; no changes in mineral content in organs; increased expression and activity of the vacuolar H+-ATPase. | Berezin et al. 2008 |
| *AtMRP7* | *Arabidopsis thaliana* | | *N. tabacum* | CaMV 35S | Cd | TP: increased tolerance to Cd; higher Cd concentration in leaf vacuoles; more efficient retention of Cd in roots | Wojas et al. 2009 |
| *AtHMA4* | *Arabidopsis thaliana* | | *N. tabacum* | CaMV 35S | Cd | TP overexpressing *AtHMA4*: enhanced Zn translocation to the shoots of TP exposed to 10 μM Zn (but not at 0.5, 100 and 200 μM Zn); plants exposed to 0.25 or 5 μM Cd: decrease in Cd uptake  TP overexpressing *AtHMA4-trunc* (lacking the C-terminal region): reduced Zn translocation to the shoot; plants exposed to 0.25 or 5 μM Cd: decrease in Cd uptake (lesser extent than TP overexpressing *AtHMA4*)  TP overexpressing *AtHMA4-C* (the C-terminal region alone): 4-fold increase in Cd and Zn concentrations in roots and shoots | Siemianowski et al. 2011 |
| *AtHMA4* | *Arabidopsis thaliana* | | *N. tabacum* | MMV | Cd | TP: >90% reduction Cd content in leaves; lower Zn content in leaves; >90% decrease in Cd root-to-shoot transfer (knockout of *HMA4.1* and *HMA4.2* alleles); one functional *HMA4* allele enables maintaining Cd level as in NT; retarded growth, necrotic lesions, altered leaf morphology and increased water content | Liedschulte et al. 2017 |
| *AtHMA4* | *Arabidopsis thaliana* | | *N. tabacum* | MMV | Cd, Zn, Fe, Mn, Cu, P | TP: reduced Cd content up to around 90%; 4- to 6-fold higher P, Fe, Mn and Cu accumulation (standard greenhouse fertilization); similar accumulation phenotype of double-mutant under field conditions (lower extent); local Zn-deficiency response in leaves; FIT1-mediated Fe-deficiency response in roots; phosphate-starvation response involving HHO2 in leaves;  TP grown on medium with high P concentration: leaf swelling and necrosis | Liedschulte et al. 2021 |
| *AtMT2b* + *AtHMA4* | *Arabidopsis thaliana* | | *N. tabacum* | CaMV 35S | Cd | TP (double transformant *AtMT2b* + *AtHMA4*): enhanced Cd tolerance; enhanced Cd and Zn root-to-shoot translocation; unchanged Zn tolerance; unchanged Cd and Zn uptake; stronger phenotype than NT or single mutants  TP single mutants (*AtMT2b* or *AtHMA4*): phenotype similar to NT plants were similar | Grispen et al. 2011 |
| *AhHMA4* | *Arabidopsis halleri* | | *N. tabacum* | *AhHMA4_prom_* | Cd, Zn | TP: differences in Cd and Zn root/shoot partitioning dependent on metal concentrations in medium; higher Zn accumulation in the upper leaves of plants grown at low Zn conditions; lower Cd accumulation  NT: differences in Cd and Zn root/shoot partitioning dependent on metal concentrations in medium | Barabasz et al. 2010 |
| *HvHMA2* | *Hordeum vulgare* | | *N. tabacum* | CaMV 35S | Cd, Zn, Fe | TP grown at 10 µM Zn or at 5 µM Cd: increased Zn and Cd root-to-shoot translocation  TP grown at 100 µM Zn: decreased Fe translocation factor; decreased Fe accumulation in shoot; decreased Zn accumulation in the roots; lower dry weight of the roots; increased Zn concentration in apoplastic fluid  TP grown at 5 µM Cd: decreased Zn and Cd accumulation in the roots (no significant changes in the shoot); increased Fe accumulation in the roots; decreased Fe accumulation in shoot  TP: increased sensitivity to Zn | Barabasz et al. 2013 |
| *OsHMA3* | *Oryza sativa* | | *N. tabacum* | *NtREL1* | Cd | TP: reduced Cd accumulation in the shoots (grown in hydroponics or soil); lower root-to-shoot translocation of Cd; decrease in oxidative stress level in the shoots | Cai et al. 2019 |
| *BjYSL7* | *Brassica juncea* | | *N. tabacum* | CaMV 35S | Cd, Ni, Fe | TP grown on medium with 50 µM CdCl_2_ or 100 µM NiCl_2_: longer roots length, lower relative inhibition rate of lengths and superior root hair development; higher Cd and Ni content in shoot; higher Fe accumulation in shoot and seeds; lower Fe accumulation in the roots | Wang et al. 2013 |
| *OsMTP1* | *Oryza sativa* | | *N. tabacum* | double CaMV 35S | Cd, As | TP upon Cd stress: reduced Cd stress-induced phytotoxic effects (growth inhibition, lipid peroxidation, and cell death); enhanced vacuolar thiol content; higher biomass growth rate (2.2-2.8-fold); higher Cd accumulation (1.96-2.22-fold)  TP upon As stress: moderate tolerance and accumulation of As. | Das et al. 2016 |
| *SmZIP* | *Salix matsudana* | | *N. tabacum* | CaMV 35S | Cd, Zn, Mn, Cu, Fe | TP exposed to Cd: higher growth rate; more vigorous phenotype; reduced Cd stress-induced phytotoxic effects; increased Cd, Zn, Mn, Fe and Cu contents in the roots, shoot and stem; increased assimilation factor; increased translocation factor; increased bioconcentration factor of Cd; Cd transport: the root epidermis 🡪 cortex 🡪 vascular cylinder 🡪 migration to the aboveground parts via the vascular cylinder; higher Cd accumulation rate in the above ground organs; Cd accumulation mainly in the leaf epidermis (less in the leaf mesophyll cells); decreased percentage of Cd in the cell wall; increased Cd content in the soluble fraction in the roots and leaves | Jiang et al. 2021 |
| *NtCBP4* | *N. tabacum* | | *N. tabacum* | CaMV 35S | Pb | TP expressing *NtCBP4DC* (a truncated version of this protein from which C-terminal with the calmodulin-binding domain and part of the putative cyclic nucleotide-binding domain was removed): improved tolerance to Pb^2+^; attenuated accumulation of Pb compared with plants expressing *NtCBP4*. | Sunkar et al. 2000 |
| *NtCBP4* | *N. tabacum* | | *Arabidopsis thaliana* | CaMV 35S | Ni, Pb | TP: enhanced tolerance to Ni; more sensitive to Pb excess;  TP grown on medium with 100 μM Ni^2+^: increased growth of the roots by 50%;  TP grown on medium with 200 μM Ni^2+^: enhanced tolerance to Ni; reduced Ni accumulation in shoot  TP grown on medium with 100 μM Pb^2+^: higher Pb accumulation in above-ground organs  TP grown on medium with 600 μM Pb^2+^: growth of the roots inhibited by 50%  NT grown on medium with 900 μM Pb^2+^: growth of the roots inhibited by 50% | Arazi et al. 1999 |
| *FRE1*,  *FRE2* | *Saccharomyces cerevisiae* | | *N. tabacum* | CaMV 35S | Fe | TP - double mutant (*FRE1* + *FRE2*): 4-fold higher root Fe(III) reduction at control conditions; more tolerant to Fe deficiency (hydroponics); higher chlorophyll content; 1.5-fold higher Fe concentrations in younger leaves  TP – single mutant *FRE2*: increased Fe(III) reduction in all lines; more tolerant to Fe deficiency (hydroponics); higher chlorophyll content; 1.5-fold higher Fe concentrations in younger leaves  TP – single mutant *FRE1*: increased Fe(III) reduction in some lines; not differences in tolerant to Fe deficiency, chlorophyll content and Fe concentrations in younger leaves  compared with NT plants  Increased Fe(III) reduction: detected along the entire length of the roots and on shoot sections | Samuelsen et al. 1998 |
| *Fic1* | *Glycine max* | | *N. tabacum* | CaMV 35S | Fe | TP: increased Fe accumulation; ferritin observed in the tissues of leaves and stems; 30% higher maximal Fe content in leaves | Goto et al. 1998 |
| *Fic1* | *Glycine max* | | *N. tabacum* | CaMV 35S | Fe | TP: illegitimate iron sequestration in leaves; behave as Fe deficient; activation of Fe transport systems; increased ferric reductase activity in the roots; increased iron content in leaves | Van Wuytswinkel et al. 1999 |
| *Fic1* | *Glycine max* | | *N. tabacum* | CaMV 35S | Cd, Zn, Fe, Mn, Cu, Ni, Pb | TP: higher abundance of Fe, Mn, Cd, Zn (higher extent), Cu, Ni, Pb (lesser extent)  Increase in Fe content in leaves was not observed in plants grown on every tested soil Concentration of P in soil impacted Fe uptake by plants | Vansuyt et al. 2000 |
| *Fic1* | *Glycine max* | | *N. tabacum* | CaMV 35S | Cd, Fe | TP grown on medium of pH≥7.0 (rhizosphere acidification): induced Fe deficiency; increased Fe accumulation  TP grown at control conditions: increased Cd phytoavailability | Sappin-Didier et al. 2003 |
| *tzn1* | *Neurospora crassa* | | *N. tabacum* | double CaMV 35S | Zn | TP: 11-fold enhanced Zn accumulation; no significant differences in Cd^2+^, Fe^2+^, Ni^2+^, Cu^2+^, Mn^2+^ and Pb^2+^ uptake | Dixit et al. 2010 |
|  | |  | | | | | |
| 1. **Heterologous expression of metal-binding peptides and proteins** | | | | | | | |
| *MT-I* | *Mus musculus* | | *N. tabacum* | CaMV 35S | Cd, Cu | TP (*N. tabacum* cv. KY 14): 24% lower Cd concentration in shoot of seedlings; 5% higher Cd concentration in the roots of seedlings; no changes in dry weights; 14% lower Cd concentration in leaf lamina (plants exposed only to endogenous soil Cd); 12% less leaves; 9% shorter; approximately 10% higher Cu concentration in the bottom (9^th^) leaf; no differences in zinc levels  TP (*N. tabacum* cv. Petit Havana): no changes in Cd concentration in shoots; 48% increase in Cd concentration in the roots; 25% higher dry weight of seedlings shoots; 26% higher dry weight of seedlings roots; little significant differences in leaf Cd content, plant height and leaf number | Yeargan et al. 1992 |
| *MT-I* | *Mus musculus* | | *N. tabacum* | CaMV 35S | Cd | TP: enhanced tolerance to Cd (unaffected growth on medium with 200 μM Cd^2+^)  NT grown on medium with 10 μM Cd^2+^: necrosis on leaves | Pan et al. 1994 |
| *ChMTII* | *Chinese hamster* | | *N. tabacum* | CaMV 35S | Cd | TP: higher dry weight of TP grown on medium with Cd (higher than NT); no changes in Cd content; increased tolerance to Cd  NT: higher dry weight of TP grown on medium with Cd | Daghan et al. 2008 |
| *ChMTII* | *Chinese hamster* | | *N. tabacum* | CaMV 35S | Pb | TP: decrease in above ground biomass production due to increase in Pb accumulation; decrease in nutrient content in leaves due to exposure to Pb excess (the highest decrease in P content); enhanced Pb accumulation in shoot  NT: decrease in above ground biomass production due to increase in Pb accumulation; decrease in nutrient content in leaves due to exposure to Pb excess (the highest decrease in P content) | Daghan et al. 2021 |
| *hMT-II* | *Homo sapiens* | | *N. tabacum* | CaMV 35S | Cd | TP: enhanced tolerance to Cd – up to 100 μM Cd^2+^ at seedling stage | Misra & Gedamu, 1989 |
| *hMT-II* | *Homo sapiens* | | *N. tabacum* | double CaMV 35S | Cd | TP: 60-70% lower Cd concentration in the shoots; reduced Cd translocation to shoot (only ~20% of Cd absorbed was translocated) | Elmayan & Tepfer, 1994 |
| *hMT-II* | *Homo sapiens* | | *N. tabacum* | double CaMV 35S | Cd | TP: decreased Cd accumulation in leaf lamina tissue by 73 % (Cd concentration in soil: 0.2 ppm). | de Borne et al. 1998 |
| *MT* | *N. glutinosa* | | *N. tabacum* | CaMV 35S | Cd | TP: 30% of TP exhibited normal growth on medium with 200 μM CdSO_4_ (shoots rooted on medium with 200 μM CdSO_4_); Cd tolerant seeds (growth on medium with 100 μM CdSO_4_)  NT: leaf chlorosis, inhibited growth and development on medium with 50 μM CdSO_4_ | Suh et al. 1998 |
| *PjMT1* | *Prosopis juliflora* | | *N. tabacum* | CaMV 35S | Cd | TP: better survival; 9-fold higher Cd accumulation in leaves; higher chlorophyll retention | Balasundaram et al. 2014 |
| *PjMT2* | *Prosopis juliflora* | | *N. tabacum* | CaMV 35S | Cd | TP: better survival; 5-fold higher Cd accumulation in leaves; higher chlorophyll retention |  |
| *SvMThis* | *Silene vulgaris* | | *N. tabacum* | CaMV 35S | Cd | TP: increased Cd accumulation in roots and leaves;  TP treated with 300 μM CdCl_2_: inhibition of photosynthesis and mobilization of the ascorbate-glutathione cycle;  TP treated with 500 μM CdCl_2_: irreversible damage of photosynthesis and oxidative stress, an appearance of a new peroxidase isoform, changes in the leaf polypeptide pattern | Gorinova et al. 2007 |
| *SaMT2* | *Sedum alfredii* | | *N. tabacum* | CaMV 35S | Cd, Zn | TP: enhanced Cd and Zn tolerance; higher Cd and Zn accumulation (in roots and shoot); higher antioxidant enzyme activities; lower accumulation of H_2_O_2_ | Zhang et al. 2014 |
| *CUP1* | *Saccharomyces cerevisiae* | | *N. tabacum* | double CaMV 35S | Cu | TP: 7-fold higher Cu accumulation in older (lower) than in young (upper) leaves; 2-3 times higher Cu content in leaves (pooled, Cu concentration in soil: 1645 ppm); no changes in Cd content in seedlings; not enhanced tolerance to Cd | Thomas et al. 2003 |
| *HisCUP1* | recombinant fusion | | *N. tabacum* | CaMV 35S | Cd | TP: up to 90% higher Cd accumulation in above-ground parts; increased translocation to shoot | Macek et al. 2002 |
| *HisCUP1* | recombinant fusion | | *N. tabacum* | CaMV 35S | Cd | TP: up to 80% higher Cd accumulation in aerial parts; higher metal content in leaves than in the roots; higher translocation factor (5.26-6.65); tolerated up to 16.2 ppm Cd in soil | Pavlíková et al. 2004a |
| *HisCUP1* | recombinant fusion | | *N. tabacum* | CaMV 35S | Cd, Zn, Ni | TP overexpressing *HisCUP*: higher Cd accumulation; increased by 90% Cd content in aboveground organs; Cd content decreased by 49% in the roots; no changes in Zn content in aboveground organs  TP overexpressing *HisGUS*: higher Ni content; accumulation of all tested metals in increased amount  TP overexpressing *GUS*: accumulation of all tested metals in increased amount | Pavlíková et al. 2004b |
| *HisCUP1* | recombinant fusion | | *N. tabacum* | CaMV 35S | Cd | TP: higher tolerance to Cd; increased Cd accumulation | Krystofova et al. 2012 |
|  | |  | | | | | |
| 1. **Tobacco engineered for overproducing of phytochelatins (PCs)** | | | | | | | |
| *AtPCS1* | *Arabidopsis thaliana* | | *N. tabacum* | CaMV 35S | Cd | TP: increased Cd accumulation in seedlings (2-fold) and adult plants; increased Cd accumulation in the roots and shoot of adult plants (linked with higher production of PCs); ~2.2-fold longer roots; no significant changes in translocation factor;  Presence of GSH in medium: enhanced root growth, tolerance and accumulation of Cd | Pomponi et al. 2006 |
| *AtPCS1* | *Arabidopsis thaliana* | | *N. tabacum* | CaMV 35S | Cd, As | TP: increased As and/or Cd accumulation in roots; increased phytochelatins (PCs) content; increased Cd vacuolar accumulation; increased capacity of detoxification of Cd and As | Zanella et al. 2016 |
| *AtPCS1* | *Arabidopsis thaliana* | | *N. tabacum* | CaMV 35S | Cd | TP overexpressing *AtPCS1*: hypersensitive to Cd while compared with NT and *CePCS* transformants; no changes in Cd accumulation; 5-fold higher PCS activity compared with WT or *CePCS* transformants; dramatic increase of γ-glutamylcysteine accumulation; strong depletion of glutathione (GSH); a temporary and moderate increase in phytochelatins (PCs) level; moderately decreased Cd-detoxification capacity (observed as lower SH:Cd ratios); higher oxidative stress (assessed by DAB staining)  TP overexpressing *CePCS*: no changes in Cd accumulation; no changes in PCS activity compared with NT; small reduction of glutathione content; γ-glutamylcysteine concentration slightly higher than in NT; a temporary and moderate increase in PCs level  Concentration of non-protein thiols (NPT) differed in plants exposed to 5 and 25 mM CdCl_2_ | Wojas et al. 2008 |
| *CePCS* | *Caenorhabditis elegans* | | *N. tabacum* | CaMV 35S | Cd |  |  |
| *TaPCS1* | *Triticum aestivum* | | *N. glauca* | CaMV 35S | Pb, Cd | TP: increased tolerance to Pb and Cd; 200% and 150% higher Pb transport to respectively the root tissues or to shoot; 2-fold higher Pb accumulation  TP grown at Pb excess: 160% longer roots, greener and higher leaves than those of NT plants | Gisbert et al. 2003 |
| *TaPCS1* | *Triticum aestivum* | | *N. glauca* | ? | Cd, Pb, Zn, Cu, Ni | TP: 6.0, 3.3, 4.8, 18.2 and 2.6 times higher accumulation in shoot of respectively Pb, Cd, Zn, Cu and Ni from polluted soil | Martínez et al. 2006 |
| *glyoxalase I* | *Brassica juncea* | | *N. tabacum* | CaMV 35S | Zn | TP: excess of Zn sequestered in the roots; increased tolerance to Zn  TP under exposure to high Zn concentrations: increase in the level of PCs; increased maintenance of GSH homeostasis; low amount of Zn accumulated in seeds | Singla-Pareek et al. 2006 |
| *glyoxalase II* | *Oryza sativa* | | *N. tabacum* | CaMV 35S | Zn |  |  |
|  | |  | | | | | |
| 1. **Tobacco engineered for overproducing enzymes involved in glutathione synthesis and sulphur utilization** | | | | | | | |
| *OAS-TL* | *Spinacia oleracea* | | *N. tabacum* | CaMV 35S | Cd, Se, Ni, Pb, Cu | TP: more tolerant to Cd, Se, Ni, Pb and Cu; higher chlorophyl content  TP grown on medium with 300 μM Cd^2+^, 500 μM Ni^2+^ or 250 μM SeO_4_^2−^: respectively 2.5-, 1.3- and 1.5-fold higher biomass; respectively 2.8-, 4.2- and 1.8-fold higher root length  TP grown on medium with 100 μM Cd^2+^: 1.4-fold higher Cd content in shoot; 4-fold lower Cd content in the roots | Kawashima et al. 2004 |
|  | |  | | | | | |
| 1. **Tobacco engineered for volatilization of pollutants** | | | | | | | |
| *merA* | bacteria | | *N. tabacum* | ? | Hg | TP: capable of converting Hg(II) taken up by roots to the Hg(0) (less toxic form) and volatilized it from the plan; could grow and flower on soil with Hg(II) concentration up to 500 ppm | Heaton et al. 1998 |
| *merA* | bacteria | | *N. tabacum* | ? | Hg | TP: removed 3-4-fold more Hg from hydroponic medium | Meagher et al. 2000 |
| *merApe9* | bacteria | | *N. tabacum* | CaMV 35S | Hg | TP: could be germinated on media supplemented with 50–350 μM Hg^2+^ (50 μM Hg^2+^ is lethal to NT seeds); increased rate of Hg volatization from solution with 25 μM Hg^2+^: (i) in the roots 9-fold, (ii) in leaves 8-fold and (iii) in stem 5-fold | He et al. 2001 |
| *merA*, *merB* | *Escherichia coli* Tn21 | | *N. tabacum* | *Prrn* | Hg | TP grown on medium with 400 μM phenyl-Hg^+^ acetate (PMA): doubled biomass yield  TP grown on medium with 100, 200 or 400 μM PMA: higher dry weight  The merAB operon was stably transformed into chloroplast genome. | Ruiz et al. 2003 |
| *merA*, *merB* | *Escherichia coli* Tn21 | | *N. tabacum* | *Prrn* | Hg | TP: good growth with Hg concentration in roots up to 2000 μg·g^-1^; accumulated both the organic and inorganic forms of Hg (concentration was higher than in the soil); more efficient absorption and translocation of organic form of Hg than inorganic-Hg (no difference in NT plants); 100-fold higher Hg accumulation in shoot; increased volatilization; increased transformation to elemental Hg | Hussein et al. 2007 |
| *merA*77*, merB* | *Escherichia coli* Tn21 | | *N. tabacum* | Act2 | Hg | TP: increased (i) resistance to phenylmercuric acetate (PMA) and HgCl_2_, (ii) the root length by 60-fold, (iii) dry weight by 17-fold | Rahman et al. 2008 |
|  | |  | | | | | |
| 1. **Tobacco engineered for overproducing rhamnolipids** | | | | | | | |
| *rhlA, rhlB* | *Pseudomonas aeruginosa* PAO1 | | *N. tabacum* | CaMV 35S | Al | TP; increased tolerance to high Al concentrations (lethal to NT tobacco). | Brichkova et al. 2007 |
|  | |  | | | | | |
| 1. **Tobacco engineered for overproducing organic acids** | | | | | | | |
| *CS* | *Pseudomonas aeruginosa* | | *N. tabacum* | ? | Al | TP: higher tolerance to Al | De la Fuente et al. 1997 |
|  | |  | | | | | |
| 1. **Tobacco engineered for higher biomass** | | | | | | | |
| *SiPf40* *(ZIP-like gene)* | *Setaria italica* | | *N. tabacum* | CaMV 35S | - | TP overexpressing *SiPf40*: faster growth than NT plants or TP with antisense gene; more branches, increased growth rate of lateral bud; darkgreen leaves; delayed flowering and senescence; in reproductive development stage: loss of apical dominance, shorter main stem than branches - cannot be distinguished by appearance; lower concentration of indole-3-acetic acid (IAA)  TP with antisense *SiPf40* gene: no significant differences in morphology during the whole developmental stage compared with NT  NT: main stem and branches could be distinguished | Liu et al. 2009 |

**References:**

1. Arazi T, Sunkar R, Kaplan B, Fromm H (1999) A tobacco plasma membrane calmodulin‐binding transporter confers Ni2+ tolerance and Pb2+ hypersensitivity in transgenic plants. Plant J 20(2):171-182. https://doi.org/10.1046/j.1365-313x.1999.00588.x
2. Balasundaram U, Venkataraman G, George S, Parida A (2014) Metallothioneins from a hyperaccumulating plant Prosopis juliflora show difference in heavy metal accumulation in transgenic tobacco. IJAEB 7(2):241-246. https://doi.org/10.1016/j.jbiotec.2008.11.008
3. Barabasz A, Krämer U, Hanikenne M, Rudzka J, Antosiewicz DM (2010) Metal accumulation in tobacco expressing Arabidopsis halleri metal hyperaccumulation gene depends on external supply. J Exp Bot 61(11):3057-3067. https://doi.org/10.1093/jxb/erq129
4. Barabasz A, Mills RF, Trojanowska E, Williams LE, Antosiewicz DM (2011) Expression of AtECA3 in tobacco modifies its responses to manganese, zinc and calcium. Environ Exp Bot 72(2):202-209. https://doi.org/10.1016/j.envexpbot.2011.03.006
5. Barabasz A, Wilkowska A, Tracz K, Ruszczyńska A, Bulska E, Mills RF et al (2013) Expression of HvHMA2 in tobacco modifies Zn–Fe–Cd homeostasis. J Plant Physiol 170(13):1176-1186. https://doi.org/10.1016/j.jplph.2013.03.018
6. Berezin I, Mizrachy-Dagry T, Brook E, Mizrahi K, Elazar M, Zhuo S. et al (2008) Overexpression of AtMHX in tobacco causes increased sensitivity to Mg 2+, Zn 2+, and Cd 2+ ions, induction of V-ATPase expression, and a reduction in plant size. Plant Cell Rep 27(5):939-949. https://doi.org/10.1007/s00299-007-0502-9
7. Brichkova GG, Shishlova AM, Maneshina TV (2007) Tolerance to aluminum in genetically modified tobacco plants. Cytol Genet 41(3):151-155. https://doi.org/10.3103/S0095452707030036
8. Cai H, Xie P, Zeng W, Zhai Z, Zhou W, Tang Z (2019) Root-specific expression of rice OsHMA3 reduces shoot cadmium accumulation in transgenic tobacco. Mol Breed 39(3):1-11. https://doi.org/10.1007/s11032-019-0964-9
9. Daghan H, Schaeffer A, Fischer R, Commandeur U (2008) Phytoextraction of cadmium from contaminated soil using transgenic tobacco plants. J Int Environ Appl Sci 3(5):336-345.
10. Daghan H, Uygur V, Abdullah EREN (2021) Lead Phytoremediation Potential of Wild Type and Transgenic Tobacco Plants. ISPEC Journal of Agricultural Sciences 5(1):168-182. https://doi.org/10.46291/ISPECJASvol5iss1pp168-182
11. Das N, Bhattacharya S, Maiti MK (2016) Enhanced cadmium accumulation and tolerance in transgenic tobacco overexpressing rice metal tolerance protein gene OsMTP1 is promising for phytoremediation. Plant Physiol Biochem 105:297-309. https://doi.org/10.1016/j.plaphy.2016.04.049
12. de Borne FD, Elmayan T, de Roton C, de Hys L, Tepfer M (1998) Cadmium partitioning in transgenic tobacco plants expressing a mammalian metallothionein gene. Mol Breed 4(2):83-90. https://doi.org/10.1023/A:1009669412489
13. De la Fuente JM, Ramı́rez-Rodrı́guez V, Cabrera-Ponce JL, Herrera-Estrella L (1997) Aluminum tolerance in transgenic plants by alteration of citrate synthesis. Science 276(5318):1566-1568. https://doi.org/10.1126/science.276.5318.1566
14. Dhankher OP, Shasti NA, Rosen BP, Fuhrmann M, Meagher RB (2003) Increased cadmium tolerance and accumulation by plants expressing bacterial arsenate reductase. New Phytol 159(2):431-441. https://doi.org/10.1046/j.1469-8137.2003.00827.x
15. Dixit P, Singh S, Vancheeswaran R, Patnala K, Eapen S (2010) Expression of a Neurospora crassa zinc transporter gene in transgenic Nicotiana tabacum enhances plant zinc accumulation without co‐transport of cadmium. Plant Cell Environ 33(10):1697-1707. https://doi.org/10.1111/j.1365-3040.2010.02174.x
16. Elmayan T, Tepfer M (1994) Synthesis of a bifunctional metallothionein/β‐glucuronidase fusion protein in transgenic tobacco plants as a means of reducing leaf cadmium levels. Plant J 6(3):433-440. https://doi.org/10.1046/j.1365-313X.1994.06030433.x
17. Gisbert C, Ros R, De Haro A, Walker DJ, Bernal MP, Serrano R, Navarro-Aviñó J (2003) A plant genetically modified that accumulates Pb is especially promising for phytoremediation. Biochem Biophys Res Commun 303(2):440-445. https://doi.org/10.1016/S0006-291X(03)00349-8
18. Gorinova N, Nedkovska M, Todorovska E, Simova-Stoilova L, Stoyanova Z, Georgieva K et al (2007) Improved phytoaccumulation of cadmium by genetically modified tobacco plants (Nicotiana tabacum L.) Physiological and biochemical response of the transformants to cadmium toxicity. Environ Pollut 145(1):161-170. https://doi.org/10.1016/j.envpol.2006.03.025
19. Goto F, Yoshihara T, Saiki H (1998) Iron accumulation in tobacco plants expressing soyabean ferritin gene. Transgenic Res 7(3):173-180. https://doi.org/10.1023/A:1008836812714
20. Grispen VM, Hakvoort HW, Bliek T, Verkleij JA, Schat H (2011) Combined expression of the Arabidopsis metallothionein MT2b and the heavy metal transporting ATPase HMA4 enhances cadmium tolerance and the root to shoot translocation of cadmium and zinc in tobacco. Environ Exp Bot, 72(1):71-76. https://doi.org/10.1016/j.envexpbot.2010.01.005
21. He YK, Sun JG, Feng XZ, Czakó M, Márton L (2001) Differential mercury volatilization by tobacco organs expressing a modified bacterial merA gene. Cell Res 11(3):231-236. https://doi.org/10.1038/sj.cr.7290091
22. Heaton AC, Rugh C L, Wang N J, Meagher R B (1998) Phytoremediation of mercury-and methylmercury-polluted soils using genetically engineered plants. J Soil Contam 7(4):497-509. https://doi.org/10.1080/10588339891334384
23. Hirschi KD, Korenkov VD, Wilganowski NL, Wagner GJ (2000) Expression of Arabidopsis CAX2 in tobacco. Altered metal accumulation and increased manganese tolerance. Plant Physiol 124(1):125-134. https://doi.org/10.1104/pp.124.1.125
24. Hussein HS, Ruiz ON, Terry N, Daniell H (2007) Phytoremediation of mercury and organomercurials in chloroplast transgenic plants: enhanced root uptake, translocation to shoots, and volatilization. Environ Sci Technol 41(24):8439-8446. https://doi.org/10.1021/es070908q
25. Jiang Y, Han J, Xue W, Wang J, Wang B, Liu L, Zou J (2021) Overexpression of SmZIP plays important roles in Cd accumulation and translocation, subcellular distribution, and chemical forms in transgenic tobacco under Cd stress. Ecotoxicol Environ Saf 214:112097. https://doi.org/10.1016/j.ecoenv.2021.112097
26. Kawashima CG, Noji M, Nakamura M, Ogra Y, Suzuki KT, Saito K (2004) Heavy metal tolerance of transgenic tobacco plants over-expressing cysteine synthase. Biotechnol Lett 26(2):153-157. https://doi.org/10.1023/B:BILE.0000012895.60773.ff
27. Korenkov V, Hirschi K, Crutchfield JD, Wagner GJ (2007) Enhancing tonoplast Cd/H antiport activity increases Cd, Zn, and Mn tolerance, and impacts root/shoot Cd partitioning in Nicotiana tabacum L. Planta 226(6):1379-1387. https://doi.org/10.1007/s00425-007-0577-0
28. Krystofova O, Zitka O, Krizkova S, Hynek D, Shestivska V, Adam V et al (2012) Accumulation of cadmium by transgenic tobacco plants (Nicotiana tabacum L.) carrying yeast metallothionein gene revealed by electrochemistry. Int J Electrochem Sci 7:886-907.
29. Liedschulte V, Laparra H, Battey JND, Schwaar JD, Broye H, Mark R et al (2017) Impairing both HMA4 homeologs is required for cadmium reduction in tobacco. Plant Cell Environ 40(3):364-377. https://doi.org/10.1111/pce.12870
30. Liedschulte V, Battey JND, Laparra H, Kleinhans S, Bovet L, Goepfert S (2021) Zinc uptake and HMA4 activity are required for micro-and macroelement balance in tobacco (Nicotiana tabacum) Phytochemistry 191:112911. https://doi.org/10.1016/j.phytochem.2021.112911
31. Liu Y, Feng X, Xu Y, Yu J, Ao G, Peng Z, Zhao Q (2009) Overexpression of millet ZIP-like gene (SiPf40) affects lateral bud outgrowth in tobacco and millet. Plant Physiol Biochem 47(11-12):1051-1060. https://doi.org/10.1016/j.plaphy.2009.08.007
32. Macek T, Macková M, Pavlíková D, Száková J, Truksa M, Singh Cundy A et al (2002) Accumulation of cadmium by transgenic tobacco. Acta Biotechnologica 22(1‐2):101-106. https://doi.org/10.1002/1521-3846(200205)22:1/2<101::AID-ABIO101>3.0.CO;2-N
33. Martínez M, Bernal P, Almela C, Vélez D, García-Agustín P, Serrano R, Navarro-Aviñó J (2006) An engineered plant that accumulates higher levels of heavy metals than Thlaspi caerulescens, with yields of 100 times more biomass in mine soils. Chemosphere 64(3):478-485. https://doi.org/10.1016/j.chemosphere.2005.10.044
34. Meagher RB (2000) Phytoremediation of toxic elemental and organic pollutants. Curr Opin Plant Biol 3(2):153-162. https://doi.org/10.1016/S1369-5266(99)00054-0
35. Misra S, Gedamu L (1989) Heavy metal tolerant transgenic Brassica napus L. and Nicotiana tabacum L. plants. Theor Appl Genet 78(2):161-168. https://doi.org/10.1007/BF00288793
36. Pan A, Yang M, Tie F, Li L, Chen Z, Ru B (1994) Expression of mouse metallothionein-I gene confers cadmium resistance in transgenic tobacco plants. Plant Mol Biol 24(2):341-351. https://doi.org/10.1007/BF00020172
37. Pavlı́ková D, Macek T, Macková M, Száková J, Balı́k J (2004a) Cadmium tolerance and accumulation in transgenic tobacco plants with a yeast metallothionein combined with a polyhistidine tail. Int Biodeterior Biodegradation 54(2-3):233-237. https://doi.org/10.1016/j.ibiod.2004.03.008
38. Pavlikova D, Macek T, Mackova M, Sura M, Szakova J, Tlustos P (2004b) The evaluation of cadmium, zinc and nickel accumulation ability of transgenic tobacco bearing different transgenes. Plant Soil Environ 50(12):513-517.
39. Pomponi M, Censi V, Di Girolamo V, De Paolis A, Di Toppi LS, Aromolo R et al (2006) Overexpression of Arabidopsis phytochelatin synthase in tobacco plants enhances Cd 2+ tolerance and accumulation but not translocation to the shoot. Planta 223(2):180-190. https://doi.org/10.1007/s00425-005-0073-3
40. Rahman RA, Abou-Shanab RA, Moawad H (2008) Mercury detoxification using genetic engineered Nicotiana tabacum. Glob NEST J 10:32-438. https://doi.org/10.30955/gnj.000489
41. Ruiz ON, Hussein HS, Terry N, Daniell H (2003) Phytoremediation of organomercurial compounds via chloroplast genetic engineering. Plant Physiol 132(3):1344-1352. https://doi.org/10.1104/pp.103.020958
42. Samuelsen AI, Martin RC, Mok DW., Mok MC (1998) Expression of the yeast FRE genes in transgenic tobacco. Plant Physiol 118(1):51-58. https://doi.org/10.1104/pp.118.1.51
43. Sappin-Didier V, Vansuyts G, Mench M, Briat JF (2005) Cadmium availability at different soil pH to transgenic tobacco overexpressing ferritin. Plant Soil 270(1):189-197. https://doi.org/10.1007/s11104-004-1494-7
44. Shaul O, Hilgemann DW, de‐Almeida‐Engler J, Van Montagu M, Inzé D, Galili G (1999) Cloning and characterization of a novel Mg2+/H+ exchanger. EMBO J 18(14):3973-3980. https://doi.org/10.1093/emboj/18.14.3973
45. Siemianowski O, Mills RF, Williams LE, Antosiewicz DM (2011) Expression of the P1B‐type ATPase AtHMA4 in tobacco modifies Zn and Cd root to shoot partitioning and metal tolerance. Plant Biotechnol J 9(1):64-74. https://doi.org/10.1111/j.1467-7652.2010.00531.x
46. Singla-Pareek SL, Yadav SK, Pareek A, Reddy MK, Sopory SK (2006) Transgenic tobacco overexpressing glyoxalase pathway enzymes grow and set viable seeds in zinc-spiked soils. Plant Physiol 140(2):613-623. https://doi.org/10.1104/pp.105.073734
47. Suh MC, Choi D, Liu JR (1998) Cadmium resistance in transgenic tobacco plants expressing the Nicotiana glutinosa L. metallothionein-like gene. Molecules & Cells, Springer Science & Business Media BV, 8(6).
48. Sunkar R, Kaplan B, Bouché N, Arazi T., Dolev D, Talke IN et al (2000) Expression of a truncated tobacco NtCBP4 channel in transgenic plants and disruption of the homologous Arabidopsis CNGC1 gene confer Pb2+ tolerance. Plant J 24(4):533-542. https://doi.org/10.1111/j.1365-313X.2000.00901.x
49. Thomas JC, Davies EC, Malick FK, Endreszl C, Williams CR, Abbas M et al (2003) Yeast metallothionein in transgenic tobacco promotes copper uptake from contaminated soils. Biotechnol Prog 19(2):273-280. https://doi.org/10.1021/bp025623q
50. Van Wuytswinkel O, Vansuyt G, Grignon N, Fourcroy P, Briat JF (1999) Iron homeostasis alteration in transgenic tobacco overexpressing ferritin. Plant J 17(1):93-97. https://doi.org/10.1046/j.1365-313X.1999.00349.x
51. Vansuyt G, Mench M, Briat JF (2000) Soil-dependent variability of leaf iron accumulation in transgenic tobacco overexpressing ferritin. Plant Physiol Biochem 38(6):499-506. https://doi.org/10.1016/S0981-9428(00)00763-4
52. Wang JW, Li Y, Zhang YX, Chai TY (2013) Molecular cloning and characterization of a Brassica juncea yellow stripe-like gene, BjYSL7, whose overexpression increases heavy metal tolerance of tobacco. Plant Cell Rep 32(5):651-662. https://doi.org/10.1007/s00299-013-1398-1
53. Wojas S, Clemens S, Hennig J, Skłodowska A, Kopera E, Schat H et al (2008) Overexpression of phytochelatin synthase in tobacco: distinctive effects of AtPCS1 and CePCS genes on plant response to cadmium. J Exp Bot 59(8):2205-2219. https://doi.org/10.1093/jxb/ern092
54. Wojas S, Hennig J, Plaza S, Geisler M, Siemianowski O, Skłodowska A et al (2009) Ectopic expression of Arabidopsis ABC transporter MRP7 modifies cadmium root-to-shoot transport and accumulation. Environ Pollut 157(10):2781-2789. https://doi.org/10.1016/j.envpol.2009.04.024
55. Yeargan R, Maiti IB, Nielsen MT, Hunt AG, Wagner GJ (1992) Tissue partitioning of cadmium in transgenic tobacco seedlings and field grown plants expressing the mouse metallothionein I gene. Transgenic Res 1(6):261-267. https://doi.org/10.1007/BF02525167
56. Zanella L, Fattorini L, Brunetti P, Roccotiello E, Cornara L, D’Angeli S et al (2016) Overexpression of AtPCS1 in tobacco increases arsenic and arsenic plus cadmium accumulation and detoxification. Planta 243(3):605-622. https://doi.org/10.1007/s00425-015-2428-8
57. Zhang J, Zhang M, Tian S, Lu L, Shohag MJI, Yang X (2014) Metallothionein 2 (SaMT2) from Sedum alfredii Hance confers increased Cd tolerance and accumulation in yeast and tobacco. PloS one 9(7):e102750. https://doi.org/10.1371/journal.pone.0102750
